# A Retrospective Analysis of Career Outcomes in Neuroscience

**DOI:** 10.1523/ENEURO.0054-24.2024

**Published:** 2024-05-24

**Authors:** Lauren E. Ullrich, John R. Ogawa, Michelle D. Jones-London

**Affiliations:** Office of Programs to Enhance the Neuroscience Workforce, National Institute of Neurological Disorders and Stroke, National Institutes of Health, Bethesda, Maryland 20892

**Keywords:** academia, career, workforce

## Abstract

What factors are associated with career outcomes among biomedical PhDs? Research to date has focused on drivers of interest in (and intention to pursue) various careers, especially during graduate school, but fewer studies have investigated participants’ ultimate career outcomes. Even less is known about what factors matter for groups historically underrepresented in the US science, technology, engineering, and mathematics (STEM) workforce, such as women, some racial and ethnic groups, and persons with disabilities ( National Center for Science and Engineering Statistics (NCSES), 2021a). This study reports a new analysis of data from 781 PhD neuroscientists that were obtained from a retrospective survey ( Ullrich et al., 2021) to investigate the factors that influence the career sector in which neuroscience PhDs are employed and whether there were group differences according to social identity. We find evidence of academia as a “default path” for incoming PhD students, but interest in different careers increases over time. Those who remained in academia had greater acceptance of the structural aspects of academic careers, such as promotion and tenure processes, and greater faculty support during postdoctoral training. Conversely, prioritizing monetary compensation and/or varied work were associated with not being in academia, while a strong interest in research was positively associated with being in nonacademic research. Somewhat surprisingly, there were few interactions with gender, and no interactions with underrepresentation status, although perhaps this was due to lower statistical power for these analyses. Our findings also underscore the role of advisors, networking, and personal relationships in securing employment in STEM.

## Significance Statement

A new analysis of a retrospective survey from 781 PhD neuroscientists who have completed their training reveals factors associated with differences between respondents who are working in different sectors, including preferences about careers and experiences in graduate and postdoctoral training. We find evidence of academia as a “default path” for incoming PhD students, but interest in different careers changes gradually over time. Our findings also underscore the role of advisors, networking, and personal relationships in securing employment in STEM. To create a more inclusive and diverse academic environment, it is essential to address financial disparities, provide tailored support, foster mentorship relationships, and actively work to create inclusive academic cultures that embrace a variety of career paths for neuroscientists.

## Introduction

What factors drive career outcomes among biomedical PhDs? Much research has focused on drivers of interest in (and intention to pursue) various careers, especially during graduate school. Academic faculty careers are often framed as the “default career” during biomedical PhD training (Sauermann and Roach, 2012), a framing evident in qualitative analysis of free-text responses to this survey (Ebrahimi et al., 2022), high levels of interest at the start of PhD training (Gibbs et al., 2014; Ullrich et al., 2021), and “excessively optimistic” beliefs about the chance of obtaining an academic career (Puljak and Sharif, 2009; Ganguli et al., 2022). However, interest in research-focused faculty careers decreases during PhD training, a trend amplified in women and members of US-based historically underrepresented (UR) racial and ethnic groups (Golde and Dore, 2004; Fuhrmann et al., 2011; Sauermann and Roach, 2012; Gibbs et al., 2014; Roach and Sauermann, 2017; but see Wood et al., 2020).

Research on the development of career interest in science, technology, engineering, and mathematics (STEM) PhD students has shown that it is a multifactorial process that evolves over time. It is influenced by factors such as confidence in one's ability as a researcher (self-efficacy), social and intellectual feelings of belonging, interactions with one's advisor, perceptions of conflicting time demands of work and family, and institutional climate or culture (Estrada et al., 2011, 2019; Gibbs and Griffin, 2013; Gazley et al., 2014; Kahn and Ginther, 2017; Hayter and Parker, 2018; Curry and DeBoer, 2020).

Fewer research studies have investigated factors related to the ultimate choice of career (as opposed to interest in or intention to pursue a career), but since career interest likely plays a role in that choice, similar factors to those mentioned above are likely to play roles. Interviews with neuroscience and psychology PhD holders underscore the role of values, passion, and life considerations (Madan, 2022, 2024a). Additional pressures such as changes in family obligations, low wages, availability of jobs, and even external biases during the job search may also influence the ultimate sector in which someone is employed (Bloch et al., 2015; Way et al., 2016; Rivera, 2017; Quadlin, 2018).

In addition, it is possible that whatever factors affect ultimate career choice might affect members of different demographic groups (e.g., women and/or UR minority groups) in different ways, resulting in the unequal representation of certain groups in STEM careers reported by the National Center for Science and Engineering Statistics (NCSES; 2021a). This unequal representation holds true in academic research specifically—and equal representation decreases at every progressive step along the academic track. Although UR racial/ethnic groups comprise 38% of the US population, they make up only 16% of the PhD recipient pool [National Center for Science and Engineering Statistics (NCSES), 2021b], 10% of current assistant professors, and 7% of tenured faculty at US medical schools (Association of American Medical Colleges, 2023). Demographic differences are also apparent in career sectors across STEM, such as government and industry (Kahn and Ginther, 2017; Mathur et al., 2018). Data from the National Science Foundation (NSF) show several differences in employment outcomes by demographic variables. In academia, women biology PhD holders are more likely than men to be employed at educational institutions other than 4-year institutions (such as community colleges or 2-year colleges) and private nonprofits [National Center for Science and Engineering Statistics (NCSES), 2021a]. Black and Hispanic biology PhD holders are more likely than non-Hispanic white and Asian biology PhD holders to be employed in government and educational institutions other than 4-year institutions [National Center for Science and Engineering Statistics (NCSES), 2021a].

We have previously reported the results of a survey of 1,479 recent neuroscience PhD graduates who had applied for or been appointed to National Institute of Neurological Disorders and Stroke (NINDS) grants (Ullrich et al., 2021). The survey investigated how career interest changed over time, and whether differences in career interests are associated with social identity, experiences in graduate school and postdoctoral training, and personal characteristics. We found evidence that individual preferences about careers in general, and academic careers specifically, predict current career interest. Despite field-specific pressures (such as disproportionate and rapid expansion of the number of neuroscience PhDs awarded; US National Science Foundation, 2016), our findings recapitulated trends found in the broader STEM workforce (Gibbs et al., 2014, 2015; Layton et al., 2016; Sinche, 2016; Lambert et al., 2020), and these findings were moderated by social identity (a combination of gender and membership in an UR minority group) and experiences in graduate school and postdoctoral training.

A subset of respondents to our previous survey had completed their training and entered the workforce. This gave us a unique opportunity to look beyond interest in a given career to ask questions about the career that respondents ultimately ended up in. In this study, we investigated which factors, including career interest, were associated with participants’ actual career choices, namely, the job sector of their current position. We consider social identity, career interest, experiences in graduate school and postdoctoral training, personal characteristics, and measures of research experience and productivity and examine their relation to career choice. It is important to note that the relations that we report are associative, since this is an exploratory retrospective study and all variables were measured at the same time.

## Materials and Methods

### Sample

The study population was composed of (1) current graduate students or recent doctoral recipients (calendar year 2008 or later), (2) US citizens or permanent residents, and (3) participants who had applied for individual NINDS funding or had been appointed to institutional NINDS training (T32) or research education grants (R25). By virtue of conducting research within the NINDS mission, all participants were considered to be neuroscientists. Participants were identified within the National Institutes of Health (NIH) database and invited by email to complete the survey during summer 2017. The survey received 2,675 responses, representing ∼36% of identified individuals and 45% of opened emails. After data cleaning, there were 2,242 complete, eligible, and unique responses. Additional information on recruitment and data collection is described in Ullrich et al. (2021). As determined by the NIH Office of Human Subjects Research, federal regulations for the protection of human subjects did not apply to this activity.

### Definitions and sample refinement

Several criteria were applied to the 2,242 responses to refine the sample for analysis. Since social identity, namely, gender and race/ethnicity, were of primary interest for this article, responses that did not include that information were excluded, leaving 2,065 responses. Second, only participants who answered that their current position was “professional in the field” were included, leaving 866 participants. This restriction meant that participants had completed their training and had chosen at least an initial career (i.e., they were not current graduate students or postdoctoral fellows). Finally, only those in science or science-related careers were retained (i.e., not unemployed or working outside of science), which led to a final total of 781 participants.

Participants included all who answered either “male” or “female” and may include transgender respondents who identify as either a man or woman. Only two participants indicated “other” and wrote in a response for gender; they were not included in the analysis due to small numbers. Respondents from white and/or Asian backgrounds are referred to as well represented (WR), while respondents from American Indian/Alaska Native, Black/African American, Hispanic/Latino, and/or Native Hawaiian/Pacific Islander backgrounds are referred to as UR, according to the NSF definition (National Science Foundation, 2015). Moving forward, this is referred to as representation status. Disability status was collected, but persons with a disability made up <3% of the final sample, so they were not included as a separate analysis group because of the small sample size.

### Survey

The survey was a 57-question instrument administered anonymously at a single point in time (Ullrich et al., 2021; Appendix 1). The questions were iteratively developed by synthesizing from several sources, conducting cognitive testing interviews, and refining language where necessary (Ullrich et al., 2021). The survey asked about respondents’ current position, demographics, career interest, experiences in graduate school and postdoctoral training, personal characteristics, and objective measures of research experience and productivity.

Respondents were asked to categorize their current position into one of the following career groups: academic position, research focus (includes physician–scientist); academic position, teaching focus; nonacademic research (e.g., research in industry, biotech, or government settings); science-related nonresearch (e.g., science outreach, communication, policy, advocacy, or administration); and other nonscience-related careers (National Institutes of Health, 2012; Gibbs et al., 2014). Nonscience-related careers had fewer than 60 cases and were not included in the analyses for this paper since they did not represent a specific career sector and represented a wide variety of professions. Respondents were also asked about their social identity, specifically gender and race/ethnicity, and other demographic information.

Respondents were asked to rate their interest in pursuing each of the above career pathways at three time points: the start of their PhD program, the end of their PhD program, and current. Interest was measured on a 4-point Likert-type scale, where 1, no interest; 2, low interest; 3, moderate interest; and 4, strong interest.

Experiences in training included various aspects of their relationship with their primary training advisor during graduate and postdoctoral training (5-point scale from “very negative” to “very positive”), sources and helpfulness of support and career advice during the graduate and postdoctoral training (4-point scale from “no guidance provided” to “very helpful”), and feelings of social and intellectual belonging to lab/research group and department/program during graduate and postdoctoral training (5-point scale from “strongly disagree” to “strongly agree”).

Personal characteristics included confidence in one's potential as an independent researcher (measured on a 5-point agreement scale where 1 was “strongly disagree” and 5 was “strongly agree”), aspects of the career or work environment most important to the respondent (choose up to top five), and features of academia that increase or decrease desire to become a faculty member (5-point scale from “greatly decrease” to “greatly increase”).

Objective measures of research experience and productivity included years of research prior to PhD program, total years of research, years to complete PhD, total time in postdoctoral training, years since PhD completion, support by NIH prior to the PhD program, first-author publication rate (first-authored publications/total years performing research), time to PhD completion, and undergraduate or doctoral degree from a top 50 research university (as measured by research and development expenditures; National Science Board, 2016).

### Analysis

This work was designed to investigate whether individual characteristics, experiences during training, and attitudes and preferences around careers are associated with the type of current position participants held. The outcome variable was nominal (type of current position) which had the following categories: research-focused academic, teaching-focused academic, nonacademic research, and science-related nonresearch.

#### General notes

All data analyses were conducted using version 4.0.5 of the R program (R Core Team, 2018). Individual packages are cited in text when referenced. Prior to any analyses, all continuous variables were visually checked for outliers by plotting to and comparing with similar curves, and any outliers were recoded to the largest/smallest value that fits the visual curve (cap method). Using this method, four observations were capped. All interactions were evaluated in the context of component main effects and all lower-level interactions and continuous variables were centered.

Definitions for small, medium, and large effect sizes for this article were generally taken from Cohen (1988): mean differences used *d* [small (*S*) ≥ 0.2, medium (*M*) ≥ 0.5, large (*L*) ≥ 0.8] and correlation coefficients and individual regression coefficients used *r* (*S* ≥ 0.1, *M* ≥ 0.3, *L* ≥ 0.6). Finally, for odds ratios, we used (rounded) Cohen's cutoffs (*S* ≥ 1.5, *M* ≥ 2.5, *L* ≥ 4.5). Significance was indicated by adjusted *p* values <0.05.

#### Data reduction

Data reduction was performed for several constructs to reduce multicollinearity, multiple comparisons, and Type I error. We used factor analysis to reduce these constructs (e.g., relationship with advisor) into latent factors.

For each analysis, the number of factors to extract was ascertained using Bayesian Information Criterion (BIC) scores computed through the very simple structure function from the psych package in R (Revelle, 2019). Then, the fa function (also from the psych package) was used to compute maximum-likelihood solutions, with oblique rotation performed using the “promax” option. Twenty-six questions were reduced to nine factor variables.

#### Changes in career interest ratings over time: repeated measures (RM) MANOVAs

Changes over time in participants’ reported interest in the four career types that might have differed by their current position were investigated through RM multivariate analysis of variances (MANOVAs). Both time (“time”: T1, T2, and T3) and interest in the different types of careers (“type”: research-focused academic, teaching-focused academic, nonacademic research; and science-related nonresearch) were within-subjects dependent variables. Between-subjects independent variables were current position, gender, and representation status. First, an omnibus RM MANOVA containing all dependent and independent variables was computed using RM from the MANOVA.RM package (Friedrich et al., 2019). *p* values were computed using 1,000 iterations of RM's Wild Bootstrap option for ANOVA-Type Statistic, and then adjusted using the procedure from Benjamini and Hochberg (1995; see below). Since the time by type by current position interaction was significant, along with several lower-order interactions, four follow-up time by type RM MANOVAs were conducted within each level of current position, again using RM. The false discovery rate (FDR) was controlled using the procedure from Benjamini and Hochberg (1995), which was applied to each analysis using the “BH” option on the mt.rawp2adjp function of R's Multtest package (Gentleman et al., 2005).

#### Gender and representation status differences in current position and explanatory variables: chi-square test, logistic regression, multinomial logistic regression, and ANOVA

Gender and representation status differences in current position and the set of 56 explanatory variables were investigated using analysis techniques that matched the nature of the dependent variables (here current position and the explanatory variables): a contingency table and chi-square for current position, logistic regression for the dichotomous explanatory variables, multinomial logistic regression for the multinomial explanatory variables, and ANOVA for continuous explanatory variables.

Gender and representation status differences on current position, a multinomial categorical variable, were investigated by computing Pearson's chi-square test of goodness of fit on the contingency table formed by crossing social identity (gender and representation status) with current position (Table 2). Follow-up chi-square statistics were computed for each subtable (within social identity type). For subtables with significant differences, standardized cell residuals (*Z*-scores) were computed for each cell.

Gender and representation status differences on explanatory variables were investigated through three different procedures, depending on the nature of the explanatory variable. For all three sets of analyses, each analysis used the explanatory variables as dependent variables and gender, representation status, and their interaction as the independent variables. The FDR was controlled using the procedure from Benjamini and Hochberg (1995), which was applied to each analysis using the “BH” option on the mt.rawp2adjp function of R's Multtest package (Pollard et al., 2005).

Gender and representation status differences on the 18 dichotomous explanatory variables were investigated through logistic regressions computed using glm from the base package in R with family as “binomial.” Gender and representation status differences on the three multinomial explanatory variables were investigated through multinomial logistic regressions computed using multinom from the nnet package in R (Venables and Ripley, 2002). Statistics for individual terms were computed by successively contrasting statistics from the full model to statistics from the three submodels that each had a different term removed using the ANOVA test for model comparison, so FDR adjustment was not possible. Finally, gender and representation status differences on the 35 continuous explanatory variables were investigated through ANOVAs computed using aov from the base package in R.

#### Two-step logistic regressions investigating factors associated with current position

Three two-step analyses were performed, one for each of the decisions presented in the Results section: (1) academia versus nonacademia, (2) research-focused academic versus teaching-focused academic, and (3) nonacademic research versus science-related, nonresearch. These branching dichotomies were chosen based on the qualitative analysis of free-text responses, which indicated that careers in academia were the “default path” for the participants (Ebrahimi et al., 2022). Each of the three analyses followed the same procedures (outlined below)—only the dependent variables changed between analyses.

Since we had 56 possible independent (explanatory) variables and two possible moderators (gender and representation status) that might be associated with each of the three dependent variables (Decisions 1 through 3), it was imperative to reduce the set of independent variables and moderation terms to only those which might be related to each of the dependent variables in a multivariate context to control both Type I error and multicollinearity. We chose to utilize a strategy first introduced by Efron et al. (2004) as the least angle regression-ordinary least squares (LARS–OLS) Hybrid and proved mathematically by Belloni and Chernozhukov (2013), in which a large set of possible independent variables is reduced using penalized regression (lasso in our case) techniques, and the resulting set of independent variables are analyzed with ordinary least squares (OLS) methods.

As an initial step in these analyses, we imputed values for any missing variables using the mice package in R (van Buuren and Groothuis-Oudshoorn, 2011). Missing values were imputed for some independent variables/predictors in order to have a full information dataset for the glinternet penalized regression procedures. Dependent variables/outcomes were not imputed. A post hoc analysis of gender and representation status showed that the group of participants who were missing data points (maximum *n* = 203) differed by 4.5 or fewer percentage points in distribution from those who were not missing data points for both gender and representation status.

The first step, lasso logistic regression, was conducted using the glinternet package in R (Lim and Hastie, 2015, 2021). The glinternet.cv procedure allows the computation of group lasso, in which groups of variables can be linked together such that their coefficients are always estimated together, an important requirement for testing interactions. It can require strong hierarchy, such that interactions are always estimated within the context of their main effects. Each of the 56 explanatory variables was crossed with gender, representation status, and the gender by representation status interaction and considered as a main effect without interaction such that the final group of variables entered into the glinternet.cv equation had 227 terms.

The gender by representation status interaction was computed separately and entered as if it was a single variable instead of an interaction term since no group lasso procedure had the capability to handle three-way interactions without including all possible interactions among independent variables, a predictor pool that would have been too large for this study. This may have led to some two-way interactions or gender and representation status main effects not being part of the three-way interaction groups, producing inflated coefficients for some of the three-way interactions. However, the only major consequence of our workaround would be that these three-way interactions were nonsignificant in the second-step OLS logistic regressions and would have effectively reduced degrees of freedom, thus making the OLS logistic regressions more conservative.

The glinternet.cv package also performs cross-validation, in which successive random subsets of the sample are analyzed leaving a specified portion of the sample (one-fifth in our case) left out for “out-of-box” testing of each model (for an overview, see James et al., 2013).

Given our interest in inference, we used a method for choosing the penalty term, lambda, that maximized coefficient size, within the generally accepted bounds of lambda.min and lambda.1se, by computing the mean coefficient size for the equation generated by each lambda between and including lambda.min and lambda.1se. We then chose the lambda that maximized coefficient size as our penalty term (Hastie et al., 2009).

Once we had chosen lambdas for each of the three lasso logistic regressions, we computed the final equations using those lambda values. These equations provided the final sets of variables that would be used in the second-step OLS logistic regressions.

The three second-step OLS logistic regressions took all variables and interactions that had nonzero coefficients in their respective first-step lasso logistic regression as their independent variables and the variable representing the decision of interest for that analysis as the dependent variable. OLS logistic regressions were performed using the glm procedure with family as “binomial” from base R. The FDR was controlled in these analyses using the procedure from Benjamini and Hochberg (1995) as outlined above.

#### Contingency table analyses investigating path to current position

The contingency table partitioned the sample into a matrix that had answers to how the participants found their current positions in rows and current position type in columns. An omnibus chi-square statistic was computed for the table to assess any association between the two variables using chisq.test in base R. We used the “simulate.*p*.value” option in chisq.test to require 2,000 Monte Carlo draws to arrive at a robust *p* value because some cells may have had low counts.

Because the omnibus chi-square test was significant, we continued with follow-up analyses to determine which rows had significantly different distributions from the overall distribution using chisq.test to compare each row to the overall row sums. Again, the “simulate.*p*.value” option was used to offset any bias from low cell counts. The *p* values for the six follow-up tests were adjusted for FDR using the procedure from Benjamini and Hochberg (1995), which was applied using the “BH” option in the adjust.*p* procedure in base R.

Finally, in rows that were found to be significantly different from the overall row distribution for the table, individual cells were compared with all other cells from the same row to identify groups of cells that were similar. We wanted to examine each method of finding a position (in rows) to find out which of the current position types (in columns) used it most often and which used it least often. Cell counts or row percentages would have been biased for this purpose by differences in the frequencies of current position types. Therefore, we compared the column percentage that was associated with each cell to the column percentages of the other cells in that row using chi-square tests of independent proportions (prop.test in base R). The FDR was controlled for these follow-up cell comparisons by adjusting the *p* values for the six follow-up tests for each row using the procedure from Benjamini and Hochberg (1995), again applied using the “BH” option in the adjust.*p* procedure in base R.

## Results

### Sample demographics

Table 1 presents basic demographic information about the 781 PhD neuroscientists in the sample. All reported that they were “professionals in the workplace,” indicating that their training was complete. Participant information includes gender (54% women, *n* = 422) and representation (WR/UR) status (16% UR, *n* = 123). Current position, the main outcome for the study, is also reported in Table 1. Slightly more than half, 51%, reported that they were in research-focused academic positions, 13% in teaching-focused academic positions, 17% in nonacademic research positions, and 19% in science-related nonresearch positions. A fifth group of 52 participants, professionals in the workplace who indicated they were currently employed in nonscience positions, were dropped from the study sample before analyses. This group was both too small to analyze with gender and representation status interactions, and also the findings would have been hard to interpret given that they did not represent a specific career sector and included a wide variety of professions. Additionally, free-text responses indicated that many respondents’ career sectors overlapped with science-related, nonresearch careers (e.g., biotechnology, data science, health care).

**Table 1.** Study sample characteristics

Demographic variable	Value	*n* (%)
Current position	Research-focused academic	396 (51%)
	Teaching-focused academic	101 (13%)
	Nonacademic research	134 (17%)
	Science-related, nonresearch	150 (19%)
Gender	Woman	422 (54%)
	Man	359 (46%)
Representation status	WR	658 (84%)
	UR	123 (16%)
Social identity	WR man	298 (38%)
	WR woman	360 (46%)
	UR man	61 (8%)
	UR woman	62 (8%)
Do you have a disability?	No	758 (97%)
	Yes	23 (3%)
First person/generation to graduate from 4-year college?	No	602 (77%)
	Yes	179 (23%)
PhD field (recoded)	Neuroscience	349 (45%)
	Biological sciences	281 (36%)
	Other sciences/engineering	76 (10%)
	Other social sciences	75 (10%)
Age range	20–24	19 (2%)
	25–29	270 (35%)
	30–34	377 (48%)
	35–39	77 (10%)
	40–44	21 (3%)
	45–50	11 (1%)
	51 or older	5 (1%)
	Prefer not to answer	1 (0%)
Median years since PhD		6
Undergraduate institution in National Science Board^a^ top 50 research university?	No	661 (85%)
	Yes	120 (15%)
Master's degree in biomedical research discipline before PhD program?	No	658 (84%)
	Yes	123 (16%)
Doctoral institution in top 50 research university?	No	383 (49%)
	Yes	398 (51%)
Ever been a postdoctoral fellow?	No	203 (26%)
	Yes	578 (74%)
Career goal changed from research-based to outside of research?	No, still is research-based	509 (65%)
	No, never was research-based	47 (6%)
	Yes, changed away from research	225 (29%)
Primary way respondent found current position (recoded)	Directly contacted by employer/recruiter	32 (4%)
	Former advisor/supervisor	79 (10%)
	Job posting	292 (37%)
	Previous position at same organization	103 (13%)
	Professional networking (other than advisor)	217 (28%)
	Other	58 (7%)

Basic demographic information about the sample of 781 PhD neuroscientists who responded to the survey and labeled themselves as currently “professionals in the field.” Totals may not equal to 100% due to rounding.

a Top 50 research university is measured by research and development expenditures (National Science Board, 2016).

In addition, 45% held a PhD in neuroscience, and the rest were in biological or health-related fields. The median years since PhD for the sample was 6, and 74% of respondents had been a postdoctoral fellow. Respondents from 193 PhD institutions in 46 states, the District of Columbia, and Puerto Rico were represented.

The sample is not a random sample but does have similar sample demographics as other studies (Gibbs et al., 2014). Due to the source for contact information (NIH's database and internet searches) and the fact that respondents had to have either applied for NIH funding or been supported by NIH funding, there is likely an overrepresentation of people in academic positions.

### Approach

After characterizing demographic information, we first investigated the relationship between career interest and the ultimate choice of career. We looked for changes in how respondents who ended up in each of the career sectors reported their career interest over time. We then investigated whether women and UR respondents reported different experiences in graduate school and postdoctoral training, personal characteristics, and objective measures of research experience and productivity when compared with men and WR respondents, respectively. Next, we investigated which of these factors were associated with participants’ current positions. Finally, we investigated whether participants’ reports of how they­ found their current positions differed by the type of position.

### Changes in career interest over time related to current position

**Figure 1.**
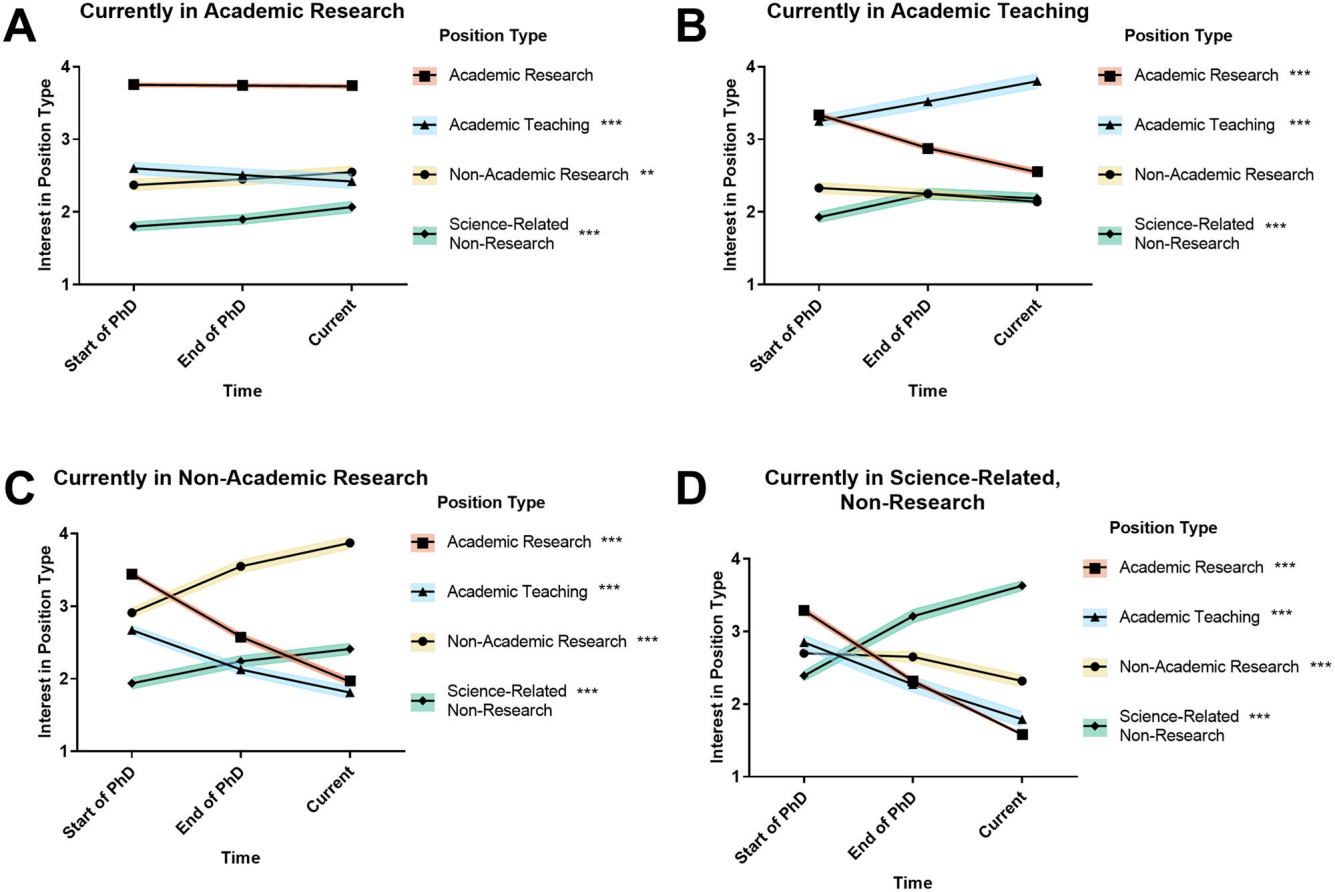
Career interest has solidified by end of PhD. Graphs show changes in career interests over time, split by current position. Mean responses of PhD neuroscientists in four different career paths who were asked to rate their level of interest in four different career paths at three time points: start of PhD, end of PhD, and current, on a 4-point scale (where 1 represents “no interest” and 4 represents “strong interest”). RM MANOVA showed that mean retrospectively reported interest in all four career types changed over time for participants in all career types except academic research-focused respondents’ interest in academic research (***A***) and academic teaching-focused respondents’ interest in nonacademic research (***B***; Extended Data Figs. 1-1–1-4; standard error around the mean is indicated by colored shading; **p < 0.01, ***p < 0.001; for *M*, SD, and effect size detail, see Extended Data Fig. 1-4).

First, we investigated whether participants’ reported interest in the four career types at three time points (start of PhD, end of PhD, and current) were related to their current positions. For example, did participants who are now in research-focused academic positions have different patterns of career interest over time than participants who are now in nonacademic research positions? To answer this question, we split participants by their current position and analyzed the change over time in their report of interest levels in various career types (Fig. 1, Extended Data Figs. 1-1–1-4).

The preliminary omnibus RM MANOVA investigating within-subjects dependent variables time and career interest rating by between-subjects independent variables current position, gender, and representation status (Extended Data Fig. 1-1) found one significant three-way interaction: current position by time by career interest rating. Follow-up time by career interest rating RM MANOVAs, split by type of current position, found significant time by career interest rating interactions for each type of current position (Extended Data Fig. 1-2). Findings were followed-up by further RM (time) ANOVAs split by current position and career interest ratings (Extended Data Fig. 1-3). These findings, along with means, are presented in Figure 1 and in Extended Data Figure 1-4.

10.1523/ENEURO.0054-24.2024.f1-1 Figure 1-1 Omnibus repeated measures MANOVA results. Results from omnibus repeated measures MANOVA to ascertain whether there were differences in the 4 Career Interest ratings over 3 Time points (within-subjects ordinal dependent variables) by Gender, UR Status, and Current Position (categorical independent variables). UR=Under-Represented, ATS BS=ANOVA-Type Statistic Bootstrap, BH Adj=Benjamini and Hochberg adjusted, Sig=Significance. *** = p < 0.001. Download Figure 1-1, DOCX file.

**Figure 2.**
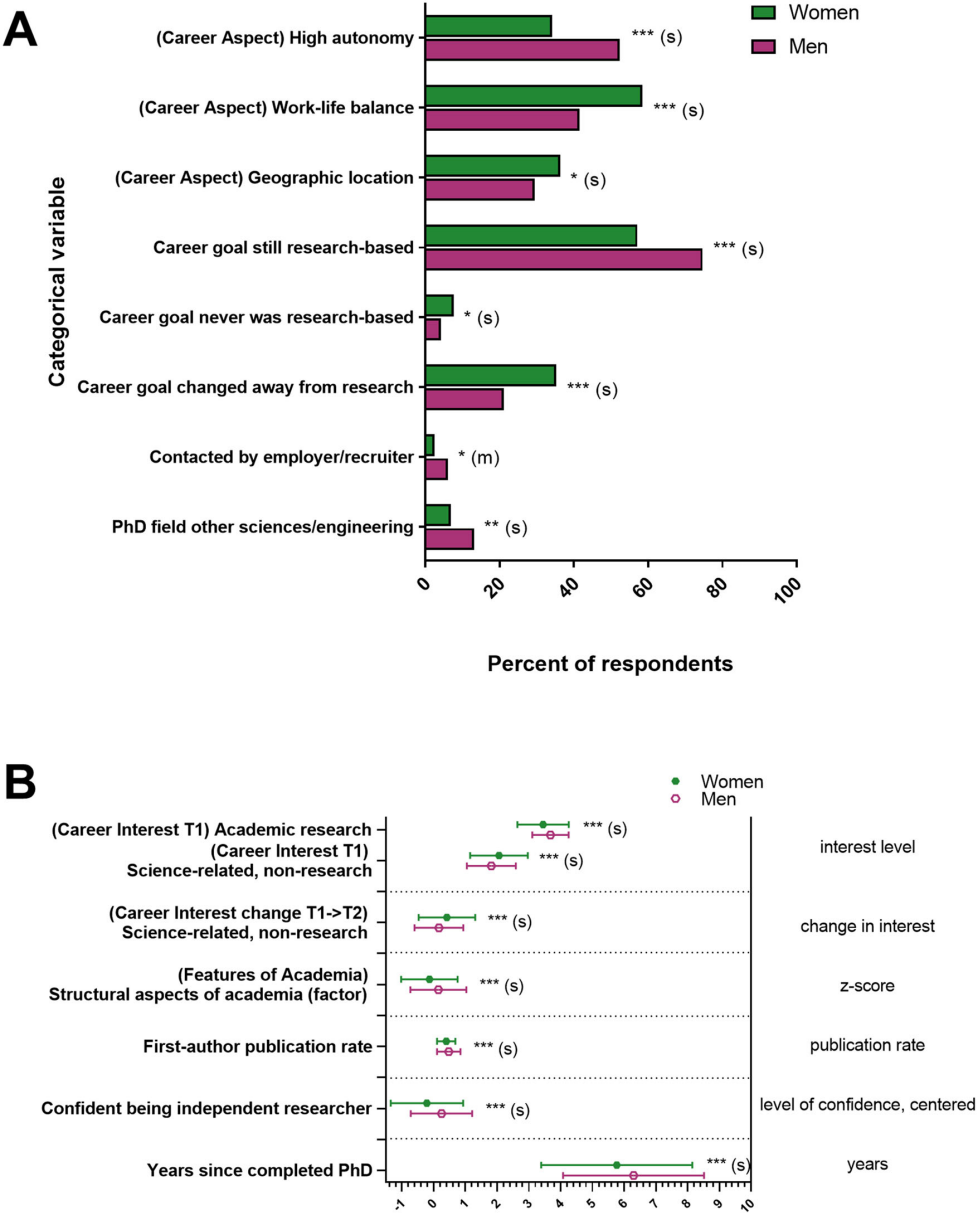
Gender differences among responses to variables capturing experiences, personal characteristics, and objective measures. Significance levels from *F* statistic (ANOVA, reported in Extended Data Figs. 2-1–2-3) comparing the means for women and men for each variable were all significant at *p* < 0.05, at least. ***A***, Categorical variables. Responses on the *x*-axis were the percent of respondents in each group who indicated either that the response was important to them or agreed with the response. ***B***, Interval variables. Responses on the *x*-axis are indicated to the right of the graph: interest levels in a given career (1–4, 4 being very interested), T2 interest minus T1 interest for change in interest, *Z*-score for “structural aspects of academia” factor scores, total publications/years of research for publication rate, level of confidence (centered −3.05 to 0.95, 0.95 being the most confident) for confidence in being an independent researcher, and years since completed PhD. Effect sizes are labeled when they reach at least “small” size. *S*, small effect size; *M*, medium effect size; *p < 0.05; **p < 0.01; ***p < 0.001. Error bars indicate standard deviation.

10.1523/ENEURO.0054-24.2024.f1-2 Figure 1-2 Four 2-way follow-up repeated measures (Time by Type of Interest) MANOVAs separately by Current Position. Results from separate repeated measures MANOVAs to ascertain whether there were differences in the 4 Career Interest ratings over 3 Time points (within-subjects ordinal dependent variables) for each of the 4 Current Positions. ATS BS=ANOVA-Type Statistic Bootstrap, BH Adj=Benjamini and Hochberg adjusted, Sig=Significance. * = p < 0.05, *** = p < 0.001. Download Figure 1-2, DOCX file.

**Figure 3.**
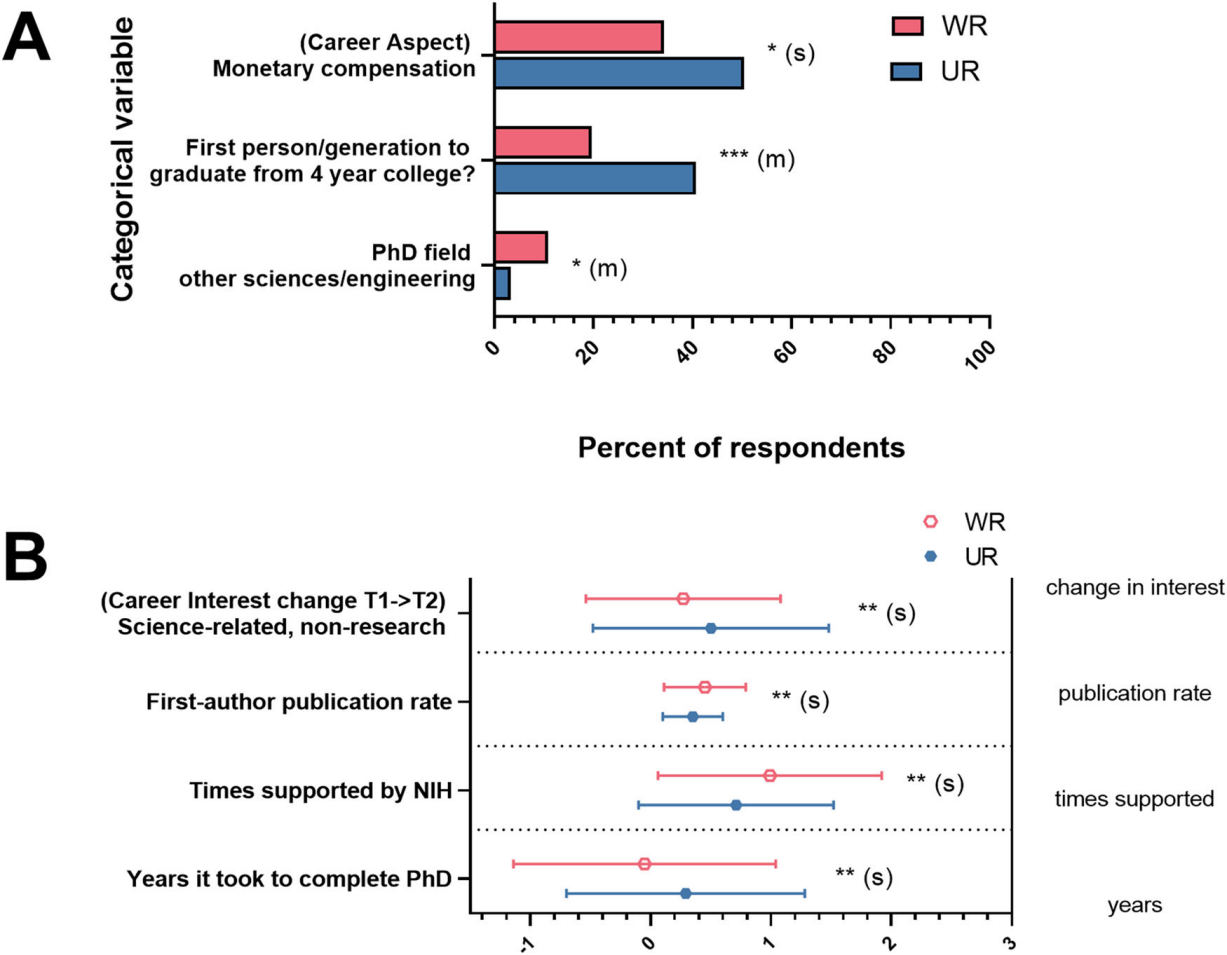
Differences between WR and UR responses to variables capturing experiences, personal characteristics, and objective measures. Significance levels from *F* statistic (ANOVA, reported in Extended Data Figs. 2-1–2-3) comparing the means for WR and UR responses for each variable were all significant at *p* < 0.05, at least. ***A***, Categorical variables. Responses on the *x*-axis were the percent of respondents in each group who indicated either that the response was important to them or agreed with the response. ***B***, Interval variables. Responses on the *x*-axis were T2 interest minus T1 interest for change in interest, total publications/years of research for publication rate, times supported by NIH, and years (centered) it took to complete PhD. Effect sizes are labeled when they reach at least “small” size. *S*, small effect size, *M*, medium effect size; *p < 0.05; **p < 0.01; ***p < 0.001. Error bars indicate standard deviation.

10.1523/ENEURO.0054-24.2024.f1-3 Figure 1-3 Sixteen 1-way follow-up repeated measures (Time) ANOVAs separately by Current Position and Type of Interest. Results from separate repeated measures ANOVAs to ascertain whether there were differences over 3 Time points (within-subjects ordinal dependent variable) for each of the 4 Current Positions and 4 Career Interest ratings. ATS BS=ANOVA-Type Statistic Bootstrap, Sig=Significance. *** = p < 0.001. Download Figure 1-3, DOCX file.

**Figure 4.**
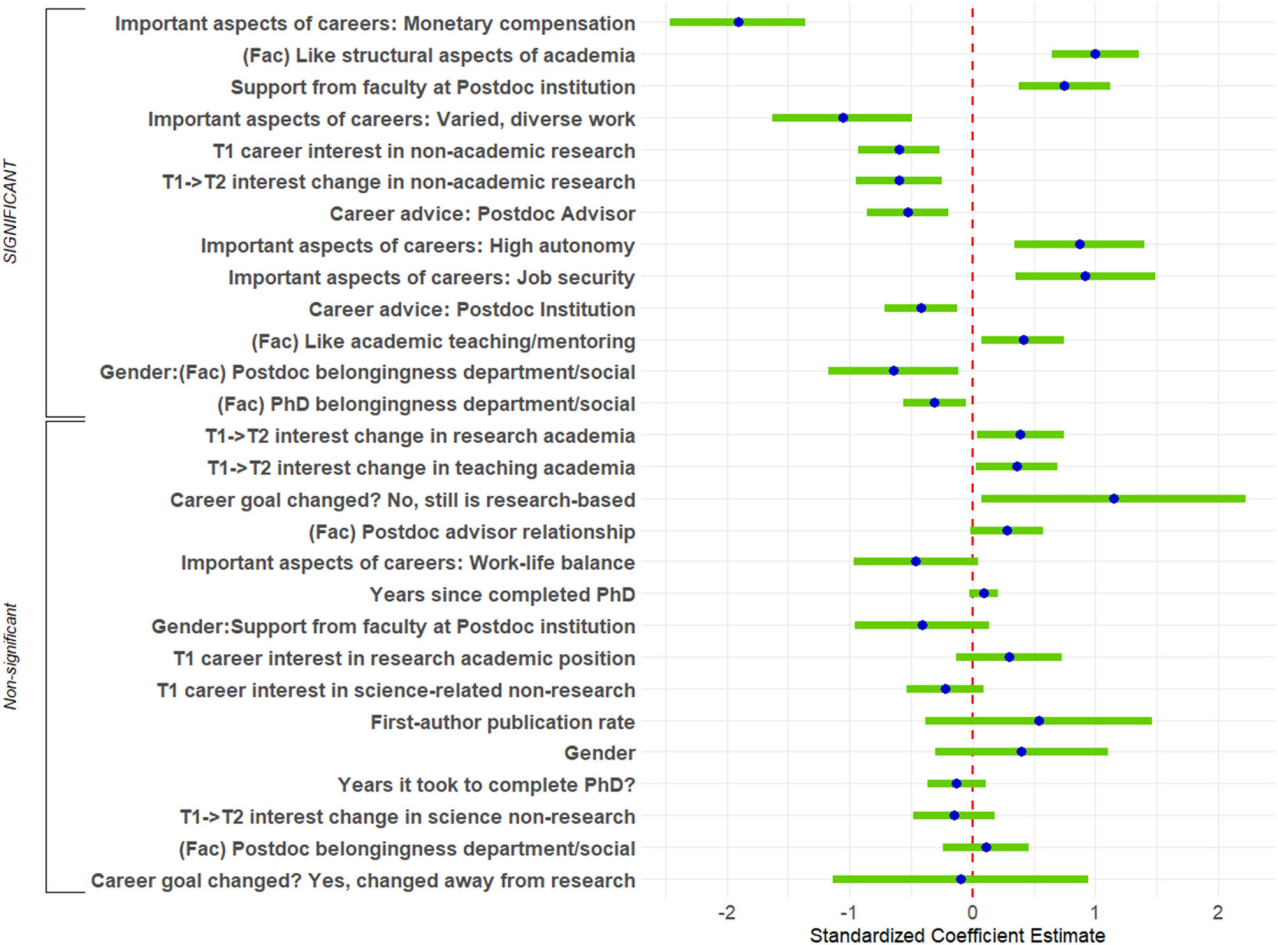
Logistic regression predicting academic versus nonacademic positions. Standardized regression coefficients and error bars for logistic regression predicting whether respondents were in academic or nonacademic positions. The dependent variable was the binary indicator of whether respondents’ positions were academic. Independent variables included career interest and change in career interest, experiences during PhD training and postdoctoral training, personal characteristics, objective measures, and interactions with gender and representation status. Factors are indicated by (Fac). The entire equation was significant at *p* < 0.001 and accurately predicted 89% of respondents in the analysis.

10.1523/ENEURO.0054-24.2024.f1-4 Figure 1-4 Mean Career Interest ratings by Current Position, Type of Interest, and Time. Means and standard deviations of Career Interest ratings with indication of significance from follow-up ANOVAs (Figure 1-3). The highest ratings for each time point are indicated by ⴕ. Sig=Significance, SD=Standard Deviation. *** = p < 0.001. Download Figure 1-4, DOCX file.

For each of the four current positions, the mean retrospectively reported interest rating changed over time for almost all career types, as demonstrated by the interest ratings of almost all types of careers, split by participants’ current positions, showing a significant within-subjects time effect (Fig. 1, Extended Data Fig. 1-4).

The first finding of note is that for those in research-focused academic positions (Fig. 1*A*), the career type with the highest mean interest rating did not change over time—it was always research-focused academic positions. In addition, while their interest in teaching-focused academic positions decreased over time, their interest in nonacademic research and science-related nonresearch increased slightly (but significantly) over time.

For participants in the three other types of positions (teaching-focused academic, nonacademic research, and scientific nonresearch), the patterns of interest were different from those of research-focused academics (Fig. 1*B–D*). For all three of the groups, the career type with the highest level of interest at the start of PhD was research-focused academia. At the end of PhD, participants report that their highest mean level of interest matched their current positions (e.g., participants who were currently nonacademic researchers report the highest mean interest in nonacademic research at the end of their PhD), often at a higher mean level than their initial interest in research-focused academia at the start of PhD. At the time of the survey (“current interest”), the career type with the highest mean level of interest remained the same as the participants’ current positions, but with an additional increase over their ratings at the end of PhD. In addition, interest in teaching-focused academic positions decreased over time for everyone except those currently in teaching-focused academic positions. In contrast, interest in science-related nonresearch positions increased for those in all four types of positions.

These patterns demonstrate that, in general, although most participants reported that they were interested in research-focused academia at the start of their PhD training (T1), by the end of their PhD training (T2), those who would end up in different career types reported that their interests had shifted to their current career type. In addition, their current interest (T3) only reinforces this shift by demonstrating an increased level of interest in their current career type.

Science-related nonresearchers showed the greatest changes in interest over time, with the greatest decrease in interest in research-focused academic positions and the greatest increase in interest in their current career type. Nonacademic researchers showed the second greatest changes in interest over time.

### Differences in current position by gender and representation status

As a prelude to analyses examining social identity and other factors that related to current position, we investigated whether there were any differences in participants’ current positions related to their social identity. Table 2 presents the results of a contingency table analysis crossing current position by social identity. The omnibus chi-square test of independence was significant (*χ*^2^_(9)_ = 70.1; *p* < 0.001), so follow-up goodness-of-fit tests (testing against the overall distribution of current positions) were performed for each social identity's distribution of current positions. Three of the four follow-ups were significant, indicating that the current position distribution for those social identities were different from the overall distribution of current positions. Finally, adjusted standardized residuals were computed for each of the three significant follow-up distributions, allowing us to determine which cells were contributing the most to the significant chi-square statistics. These residuals, and equivalent *p* values based on *Z*-score cutoffs, are displayed in the cells of Table 2.

**Table 2.** Current position by gender and representation status

Current position	Social identity				Total
	UR women***	WR women**	UR men	WR men***	
Research-focused academic	22 (35%)*	157 (44%)*	30 (49%)	187 (63%)***	396 (51%)
Teaching-focused academic	9 (14%)*	56 (16%)	8 (13%)	28 (9%)	101 (13%)
Nonacademic research	6 (10%)	54 (15%)	17 (28%)	57 (19%)	134 (17%)
Science-related, nonresearch	25 (40%)***	93 (26%)**	6 (10%)	26 (9%)***	150 (19%)
Total	62 (100%)	360 (100%)	61 (100%)	298 (100%)	781 (100%)

Proportion of “professionals in the field” respondents in each of the four “current positions” by gender and representation status. UR, underrepresented; WR, well represented. Significance level indicators (*p < 0.05, **p < 0.01, ***p < 0.001) in column headings show subset chi-square goodness-of-fit tests comparing the distribution of current positions for that social identity and the overall distribution of current positions. Significance level indicators in individual cells show *Z*-score tests for adjusted standardized residuals computed for that social identity group alone.

UR women were much more likely to be in science-related nonresearch positions and less likely to be in research-focused academic positions than the group as a whole. WR women were more likely to be in science-related nonresearch positions and less likely to be in research-focused academic positions than the group as a whole. Conversely, WR men were much more likely to be in research-focused academic positions and less likely to be in science-related nonresearch positions than the group as a whole.

### Differences in explanatory variables by gender and representation status

Next, we asked whether graduate school and postdoctoral training experiences, personal characteristics, and objective measures differed by social identity in our sample. Significant findings were followed-up by examining differences either in means or slopes for subsamples defined by whichever was significant of gender, representation status, or their interaction (Fig. 2, Extended Data Figs. 2-1–2-3).

10.1523/ENEURO.0054-24.2024.f2-1 Figure 2-1 ANOVA Results for Continuous Dependent Variables by Gender and UR Status. Results from thirty-five two-way (Gender by UR Status) ANOVAs on continuous explanatory variables (dependent variable) to ascertain whether there were Gender or UR Status differences in the explanatory variables. UR=Under-Represented, BH Adj=Benjamini and Hochberg adjusted, Sig=Significance. Effect size: (-) = negligible effect size, (S) = small effect size. * = p < 0.05, ** = p < 0.01, *** = p < 0.001. Download Figure 2-1, DOCX file.

10.1523/ENEURO.0054-24.2024.f2-2 Figure 2-2 Logistic Regression Results for Binomial Categorical Dependent Variables by Gender and UR Status. Results from eighteen two-way (Gender by UR Status) logistic regressions on dichotomous explanatory variables (dependent variable) to ascertain whether there were Gender or UR Status differences in the explanatory variables. UR=Under-Represented, BH Adj=Benjamini and Hochberg adjusted, Sig=Significance. Effect size: (-) = negligible effect size, (S) = small effect size, (M) = medium effect size. * = p < 0.05, *** = p < 0.001. Download Figure 2-2, DOCX file.

10.1523/ENEURO.0054-24.2024.f2-3 Figure 2-3 Multinomial Logistic Regression Results for Multinomial Categorical Dependent Variables by Gender and UR Status. Results from three two-way (Gender by UR Status) multinomial logistic regressions on multinomial categorical explanatory variables (dependent variable) to ascertain whether there were Gender or UR Status differences in the explanatory variables. UR=Under-Represented, Sig=Significance. * = p < 0.05, *** = p < 0.001. Download Figure 2-3, DOCX file.

#### Gender

We found significant differences in training experiences, personal characteristics, and objective metrics between men and women (significant results in Fig. 2, all results in Extended Data Figs. 2-1–2-3; all results in Fig. 2 were significantly different and had at least a small effect size).

Women were less likely to have received their PhDs in the “other sciences/engineering” category of fields than men were. Women in our sample also report they completed their PhD more recently than men and have lower publication rates than men. In addition, women's current ratings of their confidence in their potential to be independent researchers were lower than men.

Women also had different ratings than men of the importance of various aspects of careers—women rated “autonomy” less important and “work/life balance” and “geographic location” more important than men. For the factors assessing whether different features of academia increased or decreased their interest in academia, women reported that the “structural aspects of academia” factor decreased their interest in academia more than it decreased men's interest. Women were also approached directly for their current positions by employers or recruiters less than men; this was the only result that had a medium effect size.

Women's reports of their interest in different career types at the start of their graduate training also differed from men's recall of their interests. At T1, women were less interested in academic positions with a research focus and more interested in science-related nonresearch positions than men. In addition, women's interest in science-related nonresearch positions increased more over the course of their graduate training than men's interest did. Finally, women were more likely to respond that their career goals had changed away from research positions or had never been research positions compared with men.

#### Representation status

WR and UR respondents differed significantly on objective measures (significant results in Fig. 3, all results in Extended Data Figs. 2-1–2-3; all results in Fig. 3 were significantly different and had at least a small effect size). We found that UR respondents were more likely to have been supported by NIH NRSA awards fewer times before their current position than WR respondents; UR respondents took longer to obtain their PhDs; and UR respondents reported lower publication rates than WR respondents. Additionally, UR respondents were twice as likely as WR respondents to have been the first person or in the first generation of their family to graduate from a 4-year college or university and were less likely to have received their PhDs in the “other sciences/engineering” category of fields than WR respondents; both had a medium effect size.

We also found differences between UR and WR respondents in personal characteristics. UR respondents selected “monetary compensation” as an important aspect of career choice more often than WR respondents. Finally, UR respondents’ interest in science-related nonresearch positions increased more over the course of their graduate training than did WR respondent interest, even though the level of initial interest in these positions did not differ between UR and WR respondents [*t*_(161)_ = −1.28; *p*, n.s.].

There were no significant interactions between gender and representation status for any of the explanatory variables (Extended Data Figs. 2-1–2-3).

### Associations with current position

Our main question was, which factors were associated with the type of position that respondents were in currently? The analyses that addressed this question used the same broad array of explanatory variables as examined above: experiences in graduate and postdoctoral training, personal characteristics, and objective measures. In these analyses, however, they were used as independent variables along with gender and representation status and all of the interactions among them.

We analyzed our data according to the two levels of choices that mirror the decision-making process that recent PhDs and postdocs use to decide what type of jobs to pursue (this strategy of analyzing hierarchies of discrete choices has long been employed in economics through decision tree analysis; Magee, 1964). The first choice is whether to stay in academia (Helbing et al., 1998; Gaughan and Robin, 2004; Puljak and Sharif, 2009; Ebrahimi et al., 2022; Madan, 2024b). Then, for academics, the decision is between faculty positions that are research-focused and those that are teaching-focused. For nonacademics, the decision is between research positions (i.e., research in industry or government labs) and those that are science-based, but not research positions (i.e., in policy, nonprofits, or government).

We chose this two-level approach because we posit that predicting all four careers in one analysis with the entire sample might wash out similar but different associations between the outcome and the predictor variables that would be clearer when examined only for the subgroups for whom the decision mattered. For example, in the whole sample, an interest in research might predict membership in both the nonacademic research group and the research-focused academic group, which would cause any variance associated with group differences to drop out of the analysis. By separating the sample into two analyses on two different subsamples, namely, (1) determining characteristics that differentiated research versus teaching positions in only those respondents who were in academic positions and (2) determining characteristics that differentiated research versus nonresearch positions in only those respondents who were in nonacademic positions, we can capture the utility of an interest in research in differentiating the four types of positions.

#### Factors associated with being in academic versus nonacademic positions

First, we examined the associations between explanatory variables and whether respondents’ current positions were in academia or not. The details of the two-step process we used in arriving at a final logistic regression predicting academic versus nonacademic current positions are described in the Analyses section. The first step, lasso logistic regression investigating factors associated with being in academic versus nonacademic positions (Extended Data Fig. 4-1), provided the list of 28 explanatory/independent variables that were entered into the second-step OLS logistic regression. The second-step logistic regression equation (Fig. 4, Table 3) was performed on data from all 781 participants. The equation had a significant log-likelihood test, correctly predicting whether 89% of participants were in academia or were not in academia. Thirteen predictors were significant and had sufficient effect size to report.

10.1523/ENEURO.0054-24.2024.f4-1 Figure 4-1 Lasso regression predicting Academia vs. Not Academia. Results of 5-fold cross-validated lasso logistic regression predicting whether respondents were currently in academic or non-academic positions from all explanatory variables and their interactions with Gender and/or UR Status. Conf=confidence, CV=cross-validation, sd=standard deviation. Download Figure 4-1, DOCX file.

**Table 3.** Logistic regression predicting career in academia versus nonacademia

Independent variables	Unstd. coeff.	Adj. sig.	Odds ratio	Effect size	Inverse odds ratio
T1 career interest in research, academia	0.80		2.22	(*S*)	
T1 career interest in nonacademic research	0.30		1.35	-	
T1 career interest in science-related nonresearch	−0.60	**	0.55	(*S*)	1.82
(Fac) PhD belongingness department/social	−0.22		0.80	-	
T1 minus T2 interest change in research, academia	−0.31	*	0.74	-	
T1 minus T2 interest change in teaching, academia	0.39		1.47	-	
T1 minus T2 interest change in nonacademic research	0.36		1.44	-	
T1 minus T2 interest change in science nonresearch	−0.60	**	0.55	(*S*)	1.82
(Fac) Postdoc advisor relationship	−0.15		0.86	-	
Postdoc support, faculty at primary institution	0.28		1.32	-	
Postdoc career advice, advisor	−0.53	**	0.59	(*S*)	1.69
Postdoc career advice, institution	−0.42	*	0.66	(*S*)	1.52
Years it took to complete PhD?	−0.13		0.88	-	
Years since completed PhD	0.09		1.09	-	
First-author publication rate	0.54		1.72	(*S*)	
Important aspects of careers: high autonomy	0.87	**	2.38	(*S*)	
Important aspects of careers: work–life balance	−0.46		0.63	(*S*)	
Important aspects of careers: job security	0.92	**	2.51	(*M*)	
Important aspects of careers: monetary compensation	−1.91	***	0.15	(*L*)	6.67
Important aspects of careers: varied, diverse work	−1.06	**	0.35	(*M*)	2.86
(Fac) Like structural aspects of academia	1.00	***	2.72	(*M*)	
(Fac) Like academic teaching/mentoring	0.41	*	1.50	(*S*)	
Career goal changed? No, still is research-based	1.15		3.17	(*M*)	
Career goal changed? Yes, changed from research	−0.10		0.90	-	
Gender	0.40		1.50	(*S*)	
(Fac) Postdoc belongingness department/social	0.11		1.11	-	
PD support, faculty at primary institution	0.75	***	2.12	(*S*)	
Gender BY (Fac) PD belongingness department/social	−0.64	*	0.53	(*S*)	1.89
Gender BY PD support, faculty at primary institution	−0.41		0.66	(*S*)	

Results of logistic regression predicting whether respondents were in academic or nonacademic positions. Full equation statistics: *n* = 781; accuracy = 89%; likelihood ratio test *χ*^2^ = 566.29; and *p* = 0.0000. (Fac), variable is a result of factor analysis; Unstd. coeff., unstandardized coefficient; Adj. sig., FDR-adjusted significance. Effect sizes are labeled when they reach at least “small” size. *S*, small effect size; *M*, medium effect size; *L*, large effect size.

* *p* < 0.05.

** *p* < 0.01.

*** *p* < 0.001.

Several personal preferences were associated with whether participants were in academia. On the one hand, wanting job security and high autonomy were positively associated with being in academia. On the other hand, feeling that monetary compensation was an important aspect of one's career was strongly positively associated with not being in academia (large effect size), as was wanting varied/diverse work (medium effect size). Participants’ feelings about particular features of academia were also associated with being in academia, such as feeling positive about the structural aspects of academia (job market, promotion, and tenure) and feeling positive about teaching/mentoring.

Participants’ experiences as postdocs were also associated with being in academia, albeit in sometimes unintuitive ways. First, feeling that faculty support at their primary institutions was helpful was positively associated with being in academia. Rating career advice from advisors or institutions as helpful, however, was positively associated with not being in academia. Finally, feeling like one belonged to the intellectual/social community of their postdoc departments affected women and men differently (an interaction). For women, feelings of departmental belongingness were significantly positively associated with being in academia [intellectual: *χ*^2^_(4)_ = 23.87, *p* < 0.0001 and social: *χ*^2^_(4)_ = 13.12, *p* < 0.0107]. For men, feelings of belongingness were not related to being in academia.

Perhaps unsurprisingly, participants’ interest in nonacademic research positions was associated with being in nonacademic positions. High interest in nonacademic research at the start of graduate training and increases in interest in nonacademic research during graduate training were also both associated with being in nonacademic positions.

Other single variables were not reported because they were either significant but their effect sizes did not meet the threshold for reporting or had reportable effect sizes but were nonsignificant. No interactions with representation status remained significant in this analysis.

#### Factors associated with being in research-focused versus teaching-focused academic positions

**Figure 5.**
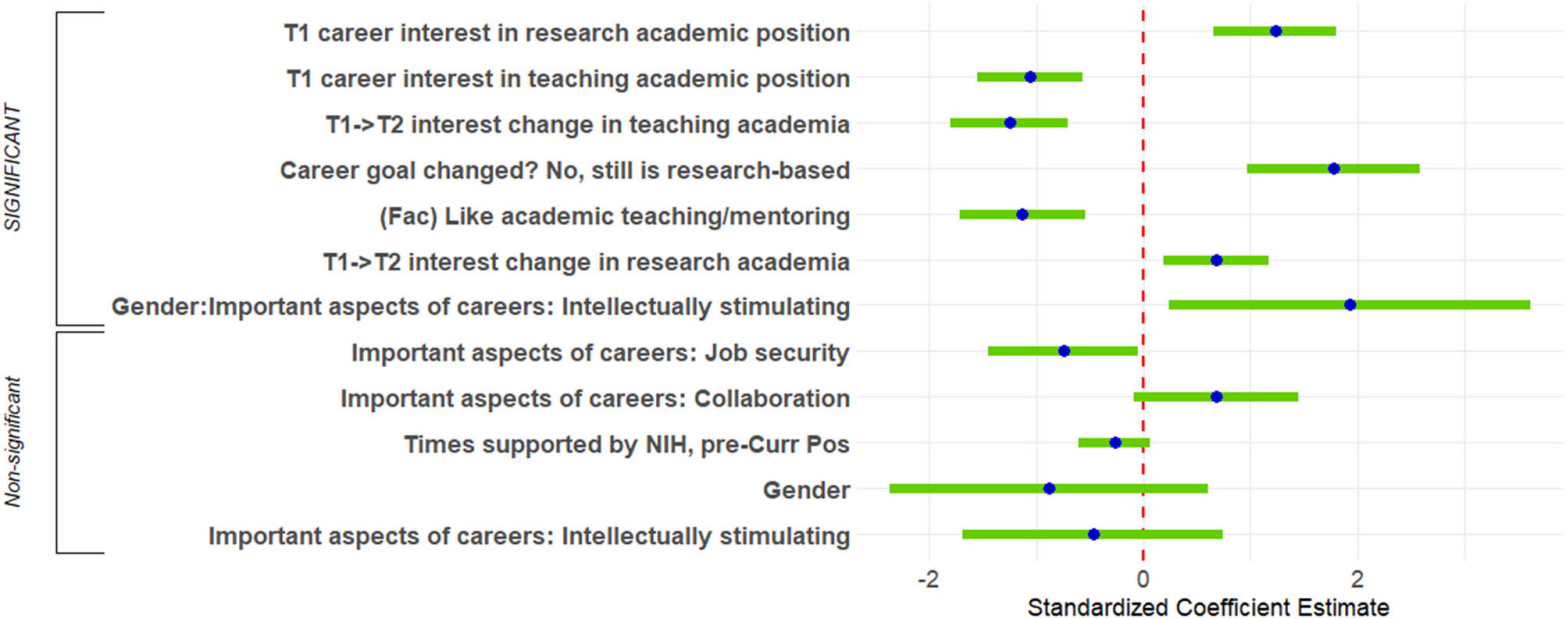
Logistic regression predicting research-focused versus teaching-focused academic positions. Standardized regression coefficients and error bars for logistic regression predicting whether respondents were in research-focused academic positions or teaching-focused academic positions. The dependent variable was the binary indicator of whether respondents’ positions were research-focused. Independent variables included career interest and change in career interest, personal characteristics, objective measures, NIH support, and interactions with gender. The entire equation was significant at *p* < 0.001 and accurately predicted 90% of respondents in the analysis. Factors are indicated by (Fac). See Extended Data Figure 5-1 for the list of explanatory/independent variables.

Next, we examined the associations between explanatory variables and respondents’ type of academic position: research- or teaching-focused. The first-step lasso logistic regression investigated factors associated with being in a research-focused academic position versus a teaching-focused academic position. Extended Data Figure 5-1 provided the list of explanatory/independent variables that were entered into the second-step OLS logistic regression. The second-step logistic regression equation (Fig. 5, Table 4) was performed on data from the 497 participants who were in academia. The equation had a significant log-likelihood test, predicting whether 90% of participants were in research-focused academic positions or teaching-focused academic positions. Seven predictors were significant and had sufficient effect size to report.

10.1523/ENEURO.0054-24.2024.f5-1 Figure 5-1 Lasso regression predicting Research Academia vs. Teaching Academia. Results of 5-fold cross-validated lasso logistic regression predicting whether respondents were currently in research or teaching academic positions from all explanatory variables and their interactions with Gender and/or UR Status. Conf=confidence, CV=cross-validation, sd=standard deviation. Download Figure 5-1, DOCX file.

**Table 4.** Logistic regression predicting research-focused versus teaching-focused academic positions

Independent variables	Unstd. coeff.	Adj. sig.	Odds ratio	Effect size	Inverse odds ratio
(Intercept)	−0.11		0.90	-	
T1 career interest in research academic	1.23	***	3.41	(*M*)	
T1 career interest in teaching academic	−1.06	***	0.35	(*M*)	2.86
T1 minus T2 interest change in research academia	0.68	*	1.97	(*S*)	
T1 minus T2 interest change in teaching academia	−1.25	***	0.29	(*M*)	3.45
Important aspects of careers: collaboration	0.68		1.97	(*S*)	
Important aspects of careers: job security	−0.75		0.47	(*S*)	
(Fac) Like academic teaching/mentoring	−1.13	***	0.32	(*M*)	3.13
Career goal changed? No, still is research-based	1.78	***	5.93	(*L*)	
Times supported by NIH, pre-Curr Pos	−0.27		0.76	-	
Gender	−0.88		0.41	(*S*)	
Aspects of careers: intellectually stimulating	−0.47		0.62	(*S*)	
Gender: aspects of careers—intellectually stimulating	1.93	*	6.90	(*L*)	

Results of logistic regression predicting whether respondents were in research-focused academic positions or teaching-focused academic positions. Full equation statistics: *n* = 497; accuracy = 90%; Akaike information criterion (AIC) = 274.75; likelihood ratio test *χ*^2^ = 253.05; and *p* = 0.0000. (Fac), variable is a result of factor analysis; Unstd. coeff., unstandardized coefficient; Adj. sig., FDR-adjusted significance. Effect sizes are labeled when they reach at least “small” size. *S*, small effect size; *M*, medium effect size; *L*, large effect size.

* *p* < 0.05.

*** *p* < 0.001.

Most of the significant predictors were strongly associated with being in research-focused academic positions and were either indicators of interest or personal preferences. Perhaps unsurprisingly, interest in research-focused academic positions at the start of graduate training was positively associated with being in research-focused academic positions, and interest in teaching-focused academic positions at the start of graduate training was positively associated with being in teaching-focused academic positions. In addition, increases in interest in research-focused academic positions over the course of graduate training were positively associated with being in research-focused academic positions, and increases in interest in teaching-focused academic positions over the course of graduate training were positively associated with being in teaching-focused academic positions.

In terms of personal preferences, choosing teaching/mentoring as a positive feature of academia was positively associated with being in a teaching-focused position, with a medium effect size. Similarly, indicating that one's “career goal was research-based and had not changed” was strongly positively associated with being in a research-focused position (large effect size).

Finally, men who chose intellectually stimulating work as an important aspect of their careers were significantly more likely than women to be in research-focused positions (89%) rather than teaching-focused positions [11%; *χ*^2^_(4)_ = 13.97; *p* < 0.0001]. For women, the association was not significant (an interaction).

#### Factors associated with being in nonacademic research versus science-related nonresearch

**Figure 6.**
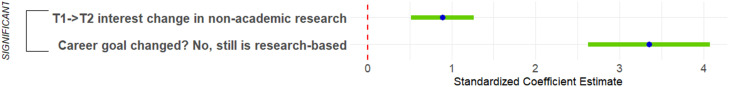
Logistic regression predicting nonacademic research versus science-related, nonresearch positions. Standardized regression coefficients and error bars for logistic regression predicting whether respondents were in nonacademic research positions or scientific nonresearch positions. The dependent variable was the binary indicator of whether respondents were in nonacademic research positions. Independent variables included change in career interest and whether respondents’ career goals had changed. The entire equation was significant at *p* < 0.001 and accurately predicted 84% of respondents in the analysis. See Extended Data Figure 6-1 for the list of explanatory/independent variables.

Finally, we examined the associations between explanatory variables and respondents’ type of nonacademic position: nonacademic research or science-related nonresearch. The first-step lasso logistic regression investigated factors associated with being in nonacademic research positions versus science-related nonresearch positions. Extended Data Figure 6-1 provides the list of explanatory/independent variables that were entered into the second-step OLS logistic regression. The second-step logistic regression equation (Table 5) was performed on data from the 284 participants who were not in academia. The equation had a significant log-likelihood test, predicting whether 84% of participants were in nonacademic research positions or science-related nonresearch positions. Only two predictors were significant and had sufficient effect size to report. As might be expected, being in nonacademic research was positively associated with both an increase in interest in nonacademic research over the course of graduate training and strongly associated with indicating that their career goal was research-based and had not changed (Fig. 6).

10.1523/ENEURO.0054-24.2024.f6-1 Figure 6-1 Lasso regression predicting Non-academic Research vs. Science/Non-research. Results of 5-fold cross-validated lasso logistic regression predicting whether respondents were currently in non-academic research or scitentific non-research positions from all explanatory variables and their interactions with Gender and/or UR Status. Conf=confidence, CV=cross-validation, sd=standard deviation. Download Figure 6-1, DOCX file.

**Table 5.** Logistic regression predicting nonacademic research versus science-related, nonresearch positions

Independent variables	Unstd. coeff.	Adj. sig.	Odds ratio	Effect size	Inverse odds ratio
(Intercept)	−1.66	***	0.19	(*L*)	5.26
T1 minus T2 interest change in nonacademic research	0.89	***	2.44	(*S*)	
Career goal changed? No, still is research-based	3.35	***	28.37	(*L*)	

Results of logistic regression predicting whether respondents were in nonacademic research positions or science-related, nonresearch positions. Full equation statistics: *n* = 284; accuracy = 84%; AIC = 240.32; likelihood ratio test *χ*^2^ = 158.48; and *p* = 0.0000. Unstd. coeff., unstandardized coefficient; Adj. sig., FDR-adjusted significance. Effect sizes are labeled when they reach at least “small” size. *S*, small effect size; *L*, large effect size.

*** *p* < 0.001.

### Differences in how participants found their current positions

The final analysis investigated whether there were any differences in the primary way participants found their current positions by type of current position. An omnibus chi-square test of independence on the contingency table was performed first and was significant (Table 6). Follow-up analyses were performed comparing types of current position for each method of finding current positions.

**Table 6.** Contingency table analysis of current position by how participants found current position

What was the primary way you found your current position?	Current position								Total (for row)		Sig. of follow-up *χ*^2^
	Research-focused academic		Teaching-focused academic		Nonacademic research		Science-related, nonresearch				
	*n*	%Col	*n*	%Col	*n*	%Col	*n*	%Col	*n*	%Col	
Contacted by employer/recruiter	13	3%	1	1%	10	8%	8	5%	32	4%	n.s.
Former advisor/supervisor	61^1^	15%	2^2^	2%	9^2^	7%	7^2^	5%	79	10%	***
Job posting	130^1^	33%	64^2^	63%	45^1^	34%	53^1^	35%	292	37%	***
Previous position at same organization	83^1^	21%	5^2^	5%	6^2^	5%	9^2^	6%	103	13%	***
Professional networking (not advisor)	76^1^	19%	28^3^	28%	56^2^	42%	57^2^	38%	217	28%	***
Other	33	8%	1	1%	8	6%	16	11%	58	7%	Not performed
Total (for column)	396	100%	101	100%	134	100%	150	100%	781	100%	***

Results of contingency table analysis relating respondents’ current positions and how they found their current positions. Rows show the distribution of people in current positions who found their positions through the primary method listed. Full table statistics: Monte Carlo simulated chi-square test of independence (2,000 runs) = 123.01 and simulated *p* = 0.0005. The total (for row) column provides the overall distribution of ways participants found their current position. Superscripts show row groups that had similar (not significantly different) rates. %Col, column percentages; Sig. of follow-up *χ*^2^ indicates FDR-adjusted significance of *χ*^2^ for that row. Superscripts represent groups (by row) that are significantly different at an FDR-corrected *p* value of 0.05 or lower.

*** *p* < 0.001.

Participants who had found their current positions through an advisor or former advisor were more likely to be in research-focused academic positions, though it was still a relatively less common way to find an academic position. Cell-by-cell follow-up comparisons showed that the percentage of time that this occurred (15%) was significantly larger than the percentage of time for all three other types of current position and that those three did not significantly differ from each other. A similar pattern occurred for participants who had found their current positions through a previous position at the same organization: participants in research-focused academic positions reported this avenue to their current positions at a higher rate (21%) than participants in all three other current positions, whose rates did not significantly differ from each other.

Another interesting finding concerned participants who had found their current positions through job postings. Although this was by far the most popular route to current positions (37% overall), it was significantly more prevalent for participants who were in teaching-focused academic positions (63%) than the three other types of current positions, whose rates did not significantly differ from each other.

Finally, those participants who had found their current positions through (nonadvisor) professional networking were more likely to be in nonacademic research positions (42%) or science-related nonresearch positions (38%; rates that did not significantly differ from each other) than participants in the other two current positions, whose rates did not significantly differ from each other. Research-focused academic positions were the least likely to have been found in this manner (19%, significantly lower than all other current position types).

## Discussion

This work was designed to retrospectively investigate the factors that influence the career sector in which neuroscience PhDs are employed, including career interests, social identity, experiences in graduate school and postdoctoral training, and personal characteristics.

This study found that the most potent predictor of career type was an individual's feelings about monetary compensation. This suggests that financial considerations play a crucial role in career decision-making for neuroscientists. It was one of several differentiators of academic versus nonacademic careers that were individual based: interests, values, and experiences. These factors reflect the personal motivations and aspirations of individuals and highlight the importance of aligning one's career with their passions and values.

Our results also point to the important role that advisors and networks had on our respondents’ career development, especially during the postdoctoral phase. Advisors not only provided valuable support but also helped their advisees secure research academic positions. Even for advisees who left academia, many of whose interests shifted gradually away from academia, advisors gave career advice that they found helpful. This underscores the significance of mentorship and guidance in shaping the career trajectories of early-career scientists. Both primary advisors and informal mentors can play a vital role in helping their mentees navigate the academic landscape and make informed career choices in the face of pervasive “unwritten rules” and the “hidden curriculum” (Pfund et al., 2016; Uddin and De Los Reyes, 2021; De Lora et al., 2022). Although no interactions with representation status remained significant in this analysis, previous research shows that science identity and feelings of belongingness are also important for the persistence of scientists from UR groups (Estrada et al., 2011; Margherio et al., 2016; Fisher et al., 2019).

### Shifting career interests

We found that, in general, although most participants reported that they were interested in research-focused academic positions at the start of their PhD training, by the end of their PhD training, those who would end up in different career types reported that their interests had shifted to their current career type. Only those currently in academic research positions reported sustained interest in that field. These results are consistent with other research indicating academic research is viewed as the default path, even by students at the start of their PhD (Helbing et al., 1998; Gaughan and Robin, 2004; Puljak and Sharif, 2009).

The continual shift in interest away from academia for those who ended up in nonacademic positions, however, indicates that respondents felt that their increased interest in nonacademic careers was not an acute event but a result of experiences, personal preferences, and mentoring up through the current time. This finding underscores the importance of mentorship and guidance for PhD students, as well as the need for exposure to a variety of career options beyond academia. It also highlights that career development is a dynamic and evolving process, and individuals may discover new interests and opportunities as they progress through their training.

### Differentiators of current position

Although many studies have investigated career interests among graduate students and postdoctoral fellows in STEM, few have information on the career sector in which respondents ultimately ended up working in. This study investigated which factors were associated with the type of position that respondents were in at the time of response.

Our approach was to organize our analyses as a series of binary questions that reflected the framing evident in qualitative analysis of free-text responses to this survey and other research (Ebrahimi et al., 2022; Madan, 2024b). We first considered, as many respondents may have, the broad choice of academia versus nonacademia and asked how participants in these two sectors differed from each other. Our findings showed that participants in academic positions (both research and teaching) valued job security and autonomy and did not prioritize monetary compensation or diverse work, compared with those in nonacademic positions. Those in academia were more positive about structural aspects of academia (job market, promotion, and tenure) and teaching/mentoring.

While many of these aspects seem inherent to an academic position, in fact they may not be, but more a consequence of tradition (Madan, 2022, 2024a). In addition, these aspects have disparate consequences depending on an individual's circumstance. In particular, not prioritizing monetary compensation may be a luxury that not all can afford. UR respondents selected “monetary compensation” as an important aspect of career choice more often than WR respondents, and our UR respondents were twice as likely as WR respondents to have been the first person or in the first generation of their family to graduate from a 4-year college or university. Levels of student loan debt and other circumstances (such as providing financial support to extended family) vary by social identity, with UR students carrying higher levels of debt (Zeiser et al., 2013; Niu, 2016; Webber and Burns, 2022). Programs such as the NIH Loan Repayment Program attempt to retain scientists in research careers through debt reduction (National Institutes of Health, n.d.).

Valuing autonomy within academia may also have disparate impacts. As a concept, autonomy is linked to an American cultural ideal in which independent, hardworking entrepreneurship leads to wealth, status, and power. Women, lower-class men, and members of UR groups face penalties when they do not appear to fulfill this model (Blair-Loy, 2013; Blair-Loy and Cech, 2022). If academic institutions wish to retain a more diverse faculty, they may wish to rethink incentive structures to encourage and recognize team science and collaboration (Cline et al., 2020).

An unspoken underlying issue for the next several findings is that, traditionally, funders, academic institutions, and faculty often consider student and postdoctoral “success” to equate to remaining in academia (Sauermann and Roach, 2012). While this attitude has been changing, institutions within the STEM ecosystem should continue to promote the broad array of careers for students and postdocs and tailor training and experiences to the career path that each individual prefers (Fuhrmann, 2016; Lenzi et al., 2020). Our findings suggest that personal history, interests, and values are all factors that contribute to an individual's career interests and outcome.

Faculty involvement in our academic respondents’ histories was a complicated picture. Faculty support during their postdoctoral training was strongly associated with remaining in academia. Respondents’ ratings of helpfulness of postdoctoral advisor career advice, however, were negatively associated with remaining in academia. It is possible that career advice as a postdoctoral fellow was more important for those who were thinking about leaving academia than those who wanted to pursue academic careers. Since postdocs as a group will generally contain both those who wish to remain in academia and those who wish to pursue other careers, academic institutions should consider fostering both faculty support of postdocs and career advising to address both groups.

Social identity was also associated with differences in respondents’ histories. For women, but not men, feelings of departmental belongingness during postdoctoral training were significantly positively associated with remaining in academia. Sense of belonging has been shown to be related to persistence in science, especially for UR groups (Luft et al., 2004; Good et al., 2012; Yen et al., 2017). The postdoctoral experience can be especially isolating, but institutions can combat this by establishing or supporting offices of postdoctoral affairs and postdoctoral associations (Åkerlind, 2005; Graham et al., 2018; Nowell et al., 2018; Fork et al., 2020).

Our second question was, if respondents remained in academia, what factors differentiated those who were in primarily research-based positions from those who were in primarily teaching-based positions? The answers were clear and perhaps unsurprising: the strongest differentiating factors were early interest (at the start of graduate school) in each type of position, becoming more interested in each type of position over the course of graduate school, and initial and sustained interest in research, which was associated with being in a research-focused position.

A more surprising finding was that choosing teaching/mentoring as a positive feature of academia was negatively associated with being in a research-focused academic position. This might not have been expected to differentiate as strongly, considering that teaching and mentorship are often considered to be a key aspect of all academic faculty positions. This aspect of the job, however, may not be as important to those who are drawn to research-focused positions. Given the interdependency between quality training and research accomplishment, these data may highlight the critical need for focused and specific mentor training built into all environments to ensure teaching and mentorship remains valued.

In addition, men, but not women, who chose “intellectually stimulating work” as an important aspect of their careers were significantly more likely to be in research-focused positions. This may indicate that women have a broader definition of what they consider “intellectually stimulating” work than men.

Our final question was, if respondents were not in academic faculty positions, what factors differentiated those who were in nonacademic research positions from those who were in science-related, nonresearch positions? The answers to this question were straightforward—a strong and/or enduring interest in research. Being in nonacademic research was positively associated with both an increase in interest in nonacademic research over the course of graduate training and strongly associated with indicating that their career goal was research-based and had not changed. No other factors differentiated these two professions, perhaps because the comparison between them was not reflective of actual choices that people might have made. Although the decision to stay in academia may be an overarching question for students and postdoctoral fellows, what to do if one leaves academia may not be as clear-cut a choice. Choosing a nonacademic career may depend on different variables than those that we measured, and it may depend on many variables, not just a few.

Interestingly, the analyses discussed above included only a few interactions with gender and no interactions with representation status. However, our results were undoubtedly limited by imbalanced group sizes, especially for analyses involving gender by representation status interactions. Since power is strongly affected by the size of the smallest subgroups (61 UR men and 62 UR women) in an analysis, these analyses may not have yielded findings because they were underpowered.

Another possibility, however, is that although there are gender and UR differences in the proportion of respondents in the different career types, the factors that led to these differences were less about how the same factors affected social identity groups differently (interactions in prediction), but that different social identity groups had different interests, values, and experiences that led to different careers. Additionally, the conditions of a given career may “select for” people who are able to succeed in that career (e.g., sectors that require unpaid internships select for those who can afford to go without pay; Kent, 2019; Hemprich-Bennett et al., 2021). This form of “survivorship bias” may limit the differences between people from UR and WR groups within the same career type.

Interestingly, whether participants had had any NIH support before their current position, which was included as a variable in the first round of all analyses, was not a significant predictor of any of the current position outcomes. In addition, because the survey was anonymous and we only collected information on prior NIH support (not unfunded applications), we could not investigate whether there was a correlation between applicants’ success in their NIH applications and career choices.

### How participants found their current positions

There were clear differences in how respondents in different types of positions found their jobs. On the one hand, those in research-focused academic positions were the most likely to have found their current positions through an advisor or former advisor or found their current positions through a previous position at the same organization. Indirect (advisors, networking) or direct (previous positions) familiarity seems to have been an important factor in hiring decisions for these positions (see similar findings among NINDS Early Stage Investigator R01 applicants in Hsu et al., 2021). Similarly, professional networking was the predominant method for those in nonacademic research positions or science-related, nonresearch positions. On the other hand, job postings, perhaps considered the default way to find a job, was the much more prevalent method for participants who were in teaching-focused academic positions—almost double the rates of the other three types of careers.

These findings underscore the role of networking and personal relationships in securing employment in STEM. Reliance on these informal and formal relationships is likely to disadvantage women and UR scientists. Research has shown that women have less access to sponsorship, support, and mentoring opportunities (Nolan et al., 2008; Moss-Racusin et al., 2012; Patton et al., 2017; Paksi and Tardos, 2021) and are less likely to benefit from networking for career enhancement (Gersick et al., 2000; Forret and Dougherty, 2004). In fact, we found that women were approached by employers or recruiters for their current positions less often than men were.

Members of UR groups also have less access to social capital through differences in size and composition of networks and strength of ties within their network (Forret, 2006). For example, Pinheiro and Melkers (2011) found that UR scientists have a greater proportion of collaborative ties outside their home institutions, but this was related to a lack of support at the home institution and chilly work climate. In Ullrich et al. (2021), we also found that UR respondents reported more beneficial relationships with faculty outside their PhD institutions than WR respondents. Projects within the NIH Neuroscience Development for Advancing the Careers of a Diverse Research Workforce R25 program support mentoring and scientific networks at a national level to address some of these factors that can isolate UR scientists and hinder their career progress (National Institute of Neurological Disorders and Stroke, 2023).

### Limitations

This study has the same limitations as discussed in Ullrich et al. (2021), which reported on respondents to the same survey: it is limited to citizens and permanent residents who have applied or been appointed to NINDS grants; it is not a random sample of respondents; it has a large number of explanatory variables; and it relies on retrospective reports. Due to the nature of the regression methods used, it also collapses rich, multifaceted social identities into large enough sample sizes to analyze, namely, binary gender and binary groups of race/ethnicity (WR and UR respondents). In addition, despite collapsing respondents into a single UR group, it is possible that our sample size was still too small to capture any but the largest differences by representation status.

Although our sample limitations restrain the breadth of the conclusions that we can draw, they are not outliers in the literature, since we have a similar sample demographic as other studies (Gibbs et al., 2014). As noted in the Materials and Methods section, we have tried to reduce the impact of the large set of explanatory variables statistically by utilizing penalized regression techniques.

Relatedly, these findings are retrospective and do not constitute independent data points. Unlike a prospective study, which would allow us to understand participants’ views at the time, retrospective ratings reflect participants’ current understanding of their interest in the past and better represent their personal narrative of their career trajectories.

A different type of limitation involves broader factors that are hard to quantify and account for. It is important to remember that although this survey is framed in terms of individual preferences and experiences, the discourse of “personal choice” can obscure the systemic pressures that different groups face (Beddoes and Pawley, 2014). For example, for women doctorates in STEM, having children and being in a dual-earner marriage are strongly associated with lower rates of labor force participation and higher rates of part-time work, likely due to women taking on the burden of family and household responsibilities (Dever et al., 2008; Schiebinger and Gilmartin, 2010; Bloch et al., 2015; Waaijer et al., 2016; Shauman, 2017; Frank, 2019).

Other factors, such as biases in hiring decisions, may also affect employment. Biases against high-performing women or assumptions around relationship or (future) parental status can introduce gender bias during hiring that may influence patterns of workforce distribution (Moss-Racusin et al., 2012; Sheltzer and Smith, 2014; Way et al., 2016; Rivera, 2017; Quadlin, 2018). Similarly, cognitive biases against UR groups have resulted in lower call-back rates during hiring (Bertrand and Mullainathan, 2003; Eaton et al., 2019; but see Lee and Savoy, 2015). These factors, although potentially strong influences on employment outcomes, are impossible to capture in studies of employees (rather than studies of employers) due to survivorship bias.

Importantly, this survey also does not reflect the effects of the COVID-19 pandemic, which are still reverberating throughout the scientific ecosystem. COVID-19 “lockdowns” in 2020 had immediate effects on scientific careers, particularly for those from marginalized identities, while the long-term effects of the pandemic on scientific careers and the scientific ecosystem remain to be seen (Gibson et al., 2020; Haas et al., 2020; Malisch et al., 2020; Doyle et al., 2021; National Academies of Sciences, 2021; Beverstock and Pickersgill, 2022; Jamali et al., 2023; Sims et al., 2023). This study captures an important snapshot from the years preceding the pandemic that future studies can be benchmarked against to understand how the pandemic has affected scientists’ career decisions.

## Conclusion

To ensure that all neuroscientists, and especially UR and/or women neuroscientists, are not driven away from academic research, but instead feel free to follow their passions (whether in academia or not), institutions and advisors will need to play a key role in reframing academic culture to value teaching, mentoring, and inclusive environments and better preparing graduate students and postdoctoral fellows for the career outcomes that match their preferences (see Ullrich et al., 2021 for discussion of potential interventions in this space). To create a more inclusive and diverse academic environment, it is essential to address financial disparities, provide tailored support, foster mentorship relationships, and actively work to create inclusive academic cultures that embrace a variety of career paths for neuroscientists.

## Synthesis

Reviewing Editor: Mark Laubach, American University Decisions are customarily a result of the Reviewing Editor and the peer reviewers coming together and discussing their recommendations until a consensus is reached. When revisions are invited, a fact-based synthesis statement explaining their decision and outlining what is needed to prepare a revision will be listed below. The following reviewer(s) agreed to reveal their identity: Lucina Uddin.

Thank you for sending your manuscript to eNeuro. The reviews are given below in full. The reviewers disagree about how we should proceed. One recommended minor revisions. The other recommended rejection. Please revise your paper thoroughly to address all concerns that were raised.

Comments from Reviewer #1

This analysis of career outcomes in neuroscience reports how interests evolve over time in PhD students, and identifies factors influencing career decisions. This is an interesting, timely report on an important topic. Some suggestions are provided below.

The authors may want to consider the data presented in these books, that engage with similar topics:

https://engra.me/books/academiabeyond/

The introduction does not mention details regarding the previous survey (how it was conducted, what were the main components, etc.)

It is not clear why the results only included those in science or science-related careers. It would be more informative to report results on all responders.

The methods section states that missing values were imputed. However, this process can be unreliable if the sample sizes are small for the participants for whom missing values are imputed. Can the authors specify exactly how many subjects (and for what demographics and what variables) missing values were imputed?

The analyses of women who are underrepresented contains very few subjects. How do discrepancies in sample sizes among the groups studied potentially affect the reported results and statistical power to detect effects of interest?

The authors may want to discuss how survivor bias factors into their null findings regarding gender and representation status.

For the discussion, the authors may want to see some recent commentaries on the importance of mentoring for demystifying the hidden curriculum of academia:

Cultivating allyship through casual mentoring to promote diversity.

Uddin LQ, De Los Reyes A.Trends Cogn Sci. 2021 Oct;25(10):813-815.

Comments from Reviewer #2

Most of the conclusions have been published by others, as evidenced by the thorough literature citing, and unfortunately potential novel ones were not addressed. For instance the study population was derived from NINDS T32 and R25 appointees and NINDS applicants. No information was provided on whether career choices correlated with funded status, or that outcomes of neuroscientists were distinguished from other biomedical/behavioral scientists. Furthermore, survey data were collected in 2017, and are outdated, esp considering the impact of the COVID interruption to many careers, as many others have shown. Lastly without having access to the earlier mentioned manuscript which used same data , it is impossible to assess duplications in these products.

## Author Response

Dear Dr. Laubach, Thank you for the opportunity to submit a revised draft of the manuscript titled, "A Retrospective Analysis of Career Outcomes in Neuroscience." We thank the reviewers for their thoughtful and insightful comments. We have incorporated changes to reflect the suggestions provided by the reviewers and have highlighted the changes within the manuscript.

Below is a point-by-point response to the reviewers' comments:

Reviewer 1 Comment: The authors may want to consider the data presented in these books, that engage with similar topics: https://engra.me/books/academiabeyond/ Response: We thank the reviewer for pointing us to these interview compilations and have incorporated them into the introduction.

Comment: The introduction does not mention details regarding the previous survey (how it was conducted, what were the main components, etc.) Response: We have added additional information about the survey into the introduction to complement the description in the methods section. Upon consultation with the reviewing editor, we have also provided the reference to the previous publication (Ullrich et al., 2021) that includes additional details on the survey.

Comment: It is not clear why the results only included those in science or science-related careers. It would be more informative to report results on all responders.

Response: Thank you for this comment. To expand on our reasoning provided in the methods section, there are a few reasons we did not include non-science positions. First, non-science positions comprise an extremely heterogeneous group of careers. These factors make interpreting findings for this group difficult, given that there would be few overall group characteristics to consider. To illustrate this point, see figure 6 and table 5, comparing non-academic research and science-related, non-research (another relatively heterogeneous group), where only two variables were significant, both related to a characteristic of the more homogeneous group (interest in research). Second, the group size for non-science positions was very small (52), making it difficult to analyze with interactions. Finally, from the open-text responses, we found that a substantial number of these respondents are employed in sectors that would be considered "science-related, non-research" (depending on job function), such as data science, biotechnology, industry, and healthcare. Because participants self-selected one of five overarching job sectors and were not asked to provide additional information about their job function or industry, we chose not to reassign respondents based on the open-text responses. Ultimately, this group is likely to overlap with the science-related, non-research group. Because of these three considerations, we did not include non-science positions in the analyses. We have revised the results text to clarify these points.

Comment: The methods section states that missing values were imputed. However, this process can be unreliable if the sample sizes are small for the participants for whom missing values are imputed. Can the authors specify exactly how many subjects (and for what demographics and what variables) missing values were imputed? Response: We have added more detail in the methods section about the missing values imputation procedures. Missing values were imputed only for some independent variables/predictors; no dependent variables/outcomes were imputed. We also added information from a post-hoc analysis which showed only <4.5 percentage points difference between participants who were missing data points and those who were not missing data points for both gender and UR status.

Comment: The analyses of women who are underrepresented contains very few subjects. How do discrepancies in sample sizes among the groups studied potentially affect the reported results and statistical power to detect effects of interest? Response: We have added a paragraph in the discussion that discusses power issues related to the underrepresented groups and the possibility that there were few significant findings because of these issues.

Comment: The authors may want to discuss how survivor bias factors into their null findings regarding gender and representation status.

Response: Thank you for this suggestion. We have added a caveat about survivorship bias into the discussion section.

Comment: For the discussion, the authors may want to see some recent commentaries on the importance of mentoring for demystifying the hidden curriculum of academia: Cultivating allyship through casual mentoring to promote diversity. Uddin LQ, De Los Reyes A.Trends Cogn Sci. 2021 Oct;25(10):813-815.

Response: We thank the reviewer for providing this reference. We have included it and other additional references to mentoring in the discussion.

Reviewer 2 Comment: Most of the conclusions have been published by others, as evidenced by the thorough literature citing, and unfortunately potential novel ones were not addressed.

Response: While the novelty of findings that came from our analyses is not within our control, we believe that both the participant population and analyses themselves are novel and there is strong justification for conducting them. We apologize for not articulating these reasons clearly and thank the reviewer for the opportunity to clarify. First, most research in this area has investigated career intention among STEM or biomedical researchers as a whole. As discussed in our previous publication, Ullrich et al., 2021, there is evidence that neuroscientists may be subject to additional or greater pressures than other biomedical scientists and STEM as a whole because of the rapid expansion of the field in recent decades. One driving reason behind our survey was to understand whether the field of neuroscience would recapitulate the trends seen in STEM fields as a whole, as reported in the literature. We have added this point to the introduction. Second, we were unable to find any quantitative manuscripts on US-based PhD students' career choices. Intention to pursue or interest in a given career is incredibly valuable information, but we believe that whether these intentions correspond to documented career outcomes is a key outstanding question in the field. The fact that our findings on career outcomes align with the qualitative literature and the quantitative research into career interest/intention (despite huge variability in sample size, methodology, and participants) only strengthens our understanding of the multifaceted process of career choice.

Comment: For instance the study population was derived from NINDS T32 and R25 appointees and NINDS applicants. No information was provided on whether career choices correlated with funded status, or that outcomes of neuroscientists were distinguished from other biomedical/behavioral scientists.

Response: We have added a paragraph in the discussion that highlights our investigation of NIH support prior to the current position, and note that it did not significantly predict the current position outcomes. We also note that, because the survey was anonymous and we only collected information on prior NIH support (not unfunded applications), we could not investigate whether there was a correlation between applicants' success in their applications and career choices.

Additionally, by virtue of applying for NINDS funding, we considered all respondents in the sample to be neuroscientists. Defining who "counts" as a neuroscientist is a potentially controversial topic (neuroscience, due to its interdisciplinary nature, cannot be captured by the official discipline of one's PhD). Our working definition of a neuroscientist is anyone who conducts neuroscience research and all research supported by NINDS falls under the broad definition of neuroscience by virtue of the NINDS mission. Therefore, we cannot directly compare outcomes of neuroscientists to other biomedical/behavioral scientists, but we do note how our findings align with the broader literature (see response above). We have added information into the methods section to clarify this.

Comment: Furthermore, survey data were collected in 2017, and are outdated, esp considering the impact of the COVID interruption to many careers, as many others have shown.

Response: We agree with this reviewer that the pandemic had extreme impacts on many careers, especially early-career researchers. However, it remains to be seen what lasting changes the pandemic will cause to both individuals' career intentions, career paths, and the scientific and academic ecosystems. We believe that our research captures an important time period pre-pandemic that can be used as a benchmark for future studies to help understand how the pandemic has changed career decisions. In addition, many of the systemic issues in science/academia remain or have even been exacerbated post-pandemic. We have added these points to the discussion.

Comment: Lastly without having access to the earlier mentioned manuscript which used same data, it is impossible to assess duplications in these products.

Response: Upon consultation with the reviewing editor, the previous manuscript has been identified and cited throughout as Ullrich et al. (2021).

We look forward to hearing from you regarding our submission and to respond to any further questions and comments you may have. Thank you.
