# Supplementary material for: A Retrospective Analysis of Career Outcomes in Neuroscience
Source: eNeuro. 2024 May 24;11(5):ENEURO.0054-24.2024. doi: 10.1523/ENEURO.0054-24.2024 (PMC11134307; doi:10.1523/ENEURO.0054-24.2024)
Supplement: Figure 1-1 — Omnibus repeated measures MANOVA results. Results from omnibus repeated measures MANOVA to ascertain whether there were differences in the 4 Career Interest ratings over 3 Time points (within-subjects ordinal dependent variables) by Gender, UR Status, and Current Position (categorical independent variables). UR=Under-Represented, ATS BS=ANOVA-Type Statistic Bootstrap, BH Adj=Benjamini and Hochberg adjusted, Sig=Significance. *** = p < 0.001. Download Figure 1-1, DOCX file. [file eneuro-11-ENEURO.0054-24.2024-s008.docx]

Figure 1-1: Omnibus repeated measures MANOVA results. Results from omnibus repeated measures MANOVA to ascertain whether there were differences in the 4 Career Interest ratings over 3 Time points (within-subjects ordinal dependent variables) by Gender, UR Status, and Current Position (categorical independent variables). UR=Under-Represented, ATS BS=ANOVA-Type Statistic Bootstrap, BH Adj=Benjamini and Hochberg adjusted, Sig=Significance. *** = p < 0.001.

| **Independent Variable(s)** | **F** | **df1** | **df2** | **Raw p** | **ATS BS p** | **BH Adj ATS BS p** | **Sig BH Adj ATS BS p** |
| --- | --- | --- | --- | --- | --- | --- | --- |
| Gender | 0.34 | 1.00 | 155.73 | 0.5615 | 0.601 | 0.81004 |  |
| UR Status | 6.08 | 1.00 | 155.73 | 0.0148 | 0.029 | 0.17980 |  |
| Gender*UR Status | 0.00 | 1.00 | 155.73 | 0.9754 | 0.980 | 0.98000 |  |
| Current Position | 0.62 | 2.58 | 155.73 | 0.5776 | 0.718 | 0.85608 |  |
| Gender*Current Position | 1.56 | 2.58 | 155.73 | 0.2080 | 0.297 | 0.78533 |  |
| UR Status*Current Position | 0.70 | 2.58 | 155.73 | 0.5355 | 0.658 | 0.83204 |  |
| Gender*UR Status*Current Position | 2.59 | 2.58 | 155.73 | 0.0640 | 0.107 | 0.41462 |  |
| Time | 1.68 | 1.92 | Inf | 0.1881 | 0.215 | 0.74056 |  |
| Gender*Time | 0.64 | 1.92 | Inf | 0.5195 | 0.546 | 0.81004 |  |
| UR Status*Time | 1.04 | 1.92 | Inf | 0.3509 | 0.363 | 0.81004 |  |
| Gender*UR Status*Time | 0.82 | 1.92 | Inf | 0.4342 | 0.480 | 0.81004 |  |
| Current Position*Time | 2.43 | 4.59 | Inf | 0.0373 | 0.036 | 0.18600 |  |
| Gender*Current Position*Time | 0.32 | 4.59 | Inf | 0.8890 | 0.913 | 0.95170 |  |
| UR Status*Current Position*Time | 0.54 | 4.59 | Inf | 0.7295 | 0.783 | 0.89900 |  |
| Gender*UR Status*Current Position*Time | 1.07 | 4.59 | Inf | 0.3746 | 0.411 | 0.81004 |  |
| Type | 95.73 | 2.68 | Inf | 0.0000 | 0.000 | 0.00000 | *** |
| Gender*Type | 4.99 | 2.68 | Inf | 0.0028 | 0.105 | 0.41462 |  |
| UR Status*Type | 1.79 | 2.68 | Inf | 0.1530 | 0.567 | 0.81004 |  |
| Gender*UR Status*Type | 1.39 | 2.68 | Inf | 0.2465 | 0.671 | 0.83204 |  |
| Current Position*Type | 39.18 | 6.74 | Inf | 0.0000 | 0.000 | 0.00000 | *** |
| Gender*Current Position*Type | 2.17 | 6.74 | Inf | 0.0362 | 0.524 | 0.81004 |  |
| UR Status*Current Position*Type | 1.17 | 6.74 | Inf | 0.3189 | 0.917 | 0.95170 |  |
| Gender*UR Status*Current Position*Type | 1.12 | 6.74 | Inf | 0.3441 | 0.921 | 0.95170 |  |
| Time*Type | 65.87 | 5.61 | Inf | 0.0000 | 0.000 | 0.00000 | *** |
| Gender*Time*Type | 1.50 | 5.61 | Inf | 0.1796 | 0.433 | 0.81004 |  |
| UR Status*Time*Type | 1.84 | 5.61 | Inf | 0.0926 | 0.285 | 0.78533 |  |
| Gender*UR Status*Time*Type | 1.15 | 5.61 | Inf | 0.3296 | 0.599 | 0.81004 |  |
| Current Position*Time*Type | 17.46 | 11.47 | Inf | 0.0000 | 0.000 | 0.00000 | *** |
| Gender*Current Position*Time*Type | 1.36 | 11.47 | Inf | 0.1784 | 0.575 | 0.81004 |  |
| UR Status*Current Position*Time*Type | 1.82 | 11.47 | Inf | 0.0423 | 0.304 | 0.78533 |  |
| Gender*UR Status*Current Position*Time*Type | 1.46 | 11.47 | Inf | 0.1348 | 0.496 | 0.81004 |  |
