# Supplementary material for: A Retrospective Analysis of Career Outcomes in Neuroscience
Source: eNeuro. 2024 May 24;11(5):ENEURO.0054-24.2024. doi: 10.1523/ENEURO.0054-24.2024 (PMC11134307; doi:10.1523/ENEURO.0054-24.2024)
Supplement: Figure 1-2 — Four 2-way follow-up repeated measures (Time by Type of Interest) MANOVAs separately by Current Position. Results from separate repeated measures MANOVAs to ascertain whether there were differences in the 4 Career Interest ratings over 3 Time points (within-subjects ordinal dependent variables) for each of the 4 Current Positions. ATS BS=ANOVA-Type Statistic Bootstrap, BH Adj=Benjamini and Hochberg adjusted, Sig=Significance. * = p < 0.05, *** = p < 0.001. Download Figure 1-2, DOCX file. [file eneuro-11-ENEURO.0054-24.2024-s009.docx]

Figure 1-2: Four 2-way follow-up repeated measures (Time by Type of Interest) MANOVAs separately by Current Position. Results from separate repeated measures MANOVAs to ascertain whether there were differences in the 4 Career Interest ratings over 3 Time points (within-subjects ordinal dependent variables) for each of the 4 Current Positions. ATS BS=ANOVA-Type Statistic Bootstrap, BH Adj=Benjamini and Hochberg adjusted, Sig=Significance. * = p < 0.05, *** = p < 0.001.

| **Current Position** | **Within-Subjects Variable(s)** | **F** | **df1** | **df2** | **Raw p** | **ATS BS p** | **BH Adj ATS BS p** | **Sig BH Adj ATS BS p** |
| --- | --- | --- | --- | --- | --- | --- | --- | --- |
| Research-focused Academic | Time | 10.04 | 1.66 | Inf | 0.000145 | 0.000 | 0.00000 | *** |
|  | Type | 626.76 | 3.34 | Inf | 0.000000 | 0.000 | 0.00000 | *** |
|  | Time*Type | 12.10 | 6.57 | Inf | 0.000000 | 0.000 | 0.00000 | *** |
| Teaching-focused Academic | Time | 0.39 | 1.93 | Inf | 0.666791 | 0.704 | 0.70400 | n.s. |
|  | Type | 130.38 | 3.36 | Inf | 0.000000 | 0.000 | 0.00000 | *** |
|  | Time*Type | 19.61 | 5.73 | Inf | 0.000000 | 0.000 | 0.00000 | *** |
| Non-academic Research | Time | 5.02 | 1.92 | Inf | 0.007335 | 0.047 | 0.04700 | * |
|  | Type | 102.61 | 2.99 | Inf | 0.000000 | 0.000 | 0.00000 | *** |
|  | Time*Type | 80.63 | 5.19 | Inf | 0.000000 | 0.000 | 0.00000 | *** |
| Scientific Non-research | Time | 25.21 | 1.68 | Inf | 0.000000 | 0.000 | 0.00000 | *** |
|  | Type | 41.18 | 3.50 | Inf | 0.000000 | 0.000 | 0.00000 | *** |
|  | Time*Type | 108.28 | 5.81 | Inf | 0.000000 | 0.000 | 0.00000 | *** |
