# Supplementary material for: A Retrospective Analysis of Career Outcomes in Neuroscience
Source: eNeuro. 2024 May 24;11(5):ENEURO.0054-24.2024. doi: 10.1523/ENEURO.0054-24.2024 (PMC11134307; doi:10.1523/ENEURO.0054-24.2024)
Supplement: Figure 1-3 — Sixteen 1-way follow-up repeated measures (Time) ANOVAs separately by Current Position and Type of Interest. Results from separate repeated measures ANOVAs to ascertain whether there were differences over 3 Time points (within-subjects ordinal dependent variable) for each of the 4 Current Positions and 4 Career Interest ratings. ATS BS=ANOVA-Type Statistic Bootstrap, Sig=Significance. *** = p < 0.001. Download Figure 1-3, DOCX file. [file eneuro-11-ENEURO.0054-24.2024-s010.docx]

Figure 1-3: Sixteen 1-way follow-up repeated measures (Time) ANOVAs separately by Current Position and Type of Interest. Results from separate repeated measures ANOVAs to ascertain whether there were differences over 3 Time points (within-subjects ordinal dependent variable) for each of the 4 Current Positions and 4 Career Interest ratings. ATS BS=ANOVA-Type Statistic Bootstrap, Sig=Significance. *** = p < 0.001.

| **Current Position** | **Career Interest Rating Type** | **F** | **df1** | **df2** | **Raw p** | **ATS BS p** | **Sig ATS BS p** |
| --- | --- | --- | --- | --- | --- | --- | --- |
| Research-focused Academic | Research-focused Academia | 0.11 | 1.78 | Inf | 0.871423 | 0.861 | n.s. |
|  | Teaching-focused Academia | 10.24 | 1.87 | Inf | 0.000057 | 0.000 | *** |
|  | Research, Non-academia | 8.56 | 1.81 | Inf | 0.000330 | 0.001 | *** |
|  | Scientific, Non-research | 23.62 | 1.75 | Inf | 0.000000 | 0.000 | *** |
| Teaching-focused Academic | Research-focused Academia | 35.10 | 1.65 | Inf | 0.000000 | 0.000 | *** |
|  | Teaching-focused Academia | 23.83 | 1.84 | Inf | 0.000000 | 0.000 | *** |
|  | Research, Non-academia | 2.06 | 1.53 | Inf | 0.139773 | 0.147 | n.s. |
|  | Scientific, Non-research | 7.51 | 1.96 | Inf | 0.000599 | 0.000 | *** |
| Non-academic Research | Research-focused Academia | 136.59 | 1.95 | Inf | 0.000000 | 0.000 | *** |
|  | Teaching-focused Academia | 56.27 | 1.82 | Inf | 0.000000 | 0.000 | *** |
|  | Research, Non-academia | 88.96 | 1.77 | Inf | 0.000000 | 0.000 | *** |
|  | Scientific, Non-research | 16.25 | 1.86 | Inf | 0.000000 | 0.000 | *** |
| Scientific Non-research | Research-focused Academia | 196.69 | 2.00 | Inf | 0.000000 | 0.000 | *** |
|  | Teaching-focused Academia | 108.84 | 1.86 | Inf | 0.000000 | 0.000 | *** |
|  | Research, Non-academia | 9.64 | 1.67 | Inf | 0.000197 | 0.000 | *** |
|  | Scientific, Non-research | 129.72 | 1.79 | Inf | 0.000000 | 0.000 | *** |
