# Supplementary material for: A Retrospective Analysis of Career Outcomes in Neuroscience
Source: eNeuro. 2024 May 24;11(5):ENEURO.0054-24.2024. doi: 10.1523/ENEURO.0054-24.2024 (PMC11134307; doi:10.1523/ENEURO.0054-24.2024)
Supplement: Figure 1-4 — Mean Career Interest ratings by Current Position, Type of Interest, and Time. Means and standard deviations of Career Interest ratings with indication of significance from follow-up ANOVAs (Figure 1-3). The highest ratings for each time point are indicated by ⴕ. Sig=Significance, SD=Standard Deviation. *** = p < 0.001. Download Figure 1-4, DOCX file. [file eneuro-11-ENEURO.0054-24.2024-s001.docx]

Figure 1-4: Mean Career Interest ratings by Current Position, Type of Interest, and Time. Means and standard deviations of Career Interest ratings with indication of significance from follow-up ANOVAs (Figure 1-3). The highest ratings for each time point are indicated by ⴕ. Sig=Significance, SD=Standard Deviation. *** = p < 0.001.

| **Current Position** | **Interest** | **Mean** | | | | **SD** | | |
| --- | --- | --- | --- | --- | --- | --- | --- | --- |
|  |  | **Sig** | Start of PhD | End of PhD | Current | Start of PhD | End of PhD | Current |
| Research-focused Academic | Research-focused Academia | n.s. | 3.75ⴕ | 3.74ⴕ | 3.73ⴕ | 0.55 | 0.59 | 0.61 |
|  | Teaching-focused Academia | *** | 2.6 | 2.51 | 2.42 | 0.83 | 0.90 | 0.98 |
|  | Research, Non-academia | *** | 2.37 | 2.45 | 2.55 | 0.90 | 0.94 | 0.96 |
|  | Scientific, Non-research | *** | 1.8 | 1.9 | 2.07 | 0.77 | 0.85 | 0.95 |
| Teaching-focused Academic | Research-focused Academia | *** | 3.34ⴕ | 2.88 | 2.55 | 0.67 | 0.75 | 0.88 |
|  | Teaching-focused Academia | *** | 3.25 | 3.52ⴕ | 3.8ⴕ | 0.82 | 0.98 | 1.03 |
|  | Research, Non-academia | n.s. | 2.33 | 2.25 | 2.14 | 0.84 | 0.74 | 0.51 |
|  | Scientific, Non-research | *** | 1.93 | 2.25 | 2.19 | 0.90 | 0.93 | 0.86 |
| Non-academic Research | Research-focused Academia | *** | 3.44ⴕ | 2.58 | 1.96 | 0.74 | 0.98 | 1.01 |
|  | Teaching-focused Academia | *** | 2.67 | 2.13 | 1.81 | 0.65 | 0.74 | 0.87 |
|  | Research, Non-academia | *** | 2.91 | 3.55ⴕ | 3.87ⴕ | 0.77 | 1.02 | 1.02 |
|  | Scientific, Non-research | *** | 1.94 | 2.24 | 2.41 | 0.95 | 0.97 | 0.95 |
| Scientific Non-research | Research-focused Academia | *** | 3.29ⴕ | 2.32 | 1.58 | 0.95 | 0.72 | 0.38 |
|  | Teaching-focused Academia | *** | 2.85 | 2.27 | 1.79 | 0.96 | 1.09 | 1.08 |
|  | Research, Non-academia | *** | 2.7 | 2.65 | 2.32 | 0.68 | 0.95 | 0.98 |
|  | Scientific, Non-research | *** | 2.39 | 3.21ⴕ | 3.63ⴕ | 0.86 | 1.15 | 0.85 |
