# Supplementary material for: A Retrospective Analysis of Career Outcomes in Neuroscience
Source: eNeuro. 2024 May 24;11(5):ENEURO.0054-24.2024. doi: 10.1523/ENEURO.0054-24.2024 (PMC11134307; doi:10.1523/ENEURO.0054-24.2024)
Supplement: Figure 2-1 — ANOVA Results for Continuous Dependent Variables by Gender and UR Status. Results from thirty-five two-way (Gender by UR Status) ANOVAs on continuous explanatory variables (dependent variable) to ascertain whether there were Gender or UR Status differences in the explanatory variables. UR=Under-Represented, BH Adj=Benjamini and Hochberg adjusted, Sig=Significance. Effect size: (-) = negligible effect size, (S) = small effect size. * = p < 0.05, ** = p < 0.01, *** = p < 0.001. Download Figure 2-1, DOCX file. [file eneuro-11-ENEURO.0054-24.2024-s002.docx]

Figure 2-1: ANOVA Results for Continuous Dependent Variables by Gender and UR Status. Results from thirty-five two-way (Gender by UR Status) ANOVAs on continuous explanatory variables (dependent variable) to ascertain whether there were Gender or UR Status differences in the explanatory variables. UR=Under-Represented, BH Adj=Benjamini and Hochberg adjusted, Sig=Significance. Effect size: (-) = negligible effect size, (S) = small effect size. * = p < 0.05, ** = p < 0.01, *** = p < 0.001.

| **(ANOVA Terms grouped by analysis) Dependent Variable: Independent Variable(s) (degrees of freedon)** | **Coefficient** | **Omega^2^** | **F** | **Raw p Value** | **BH Adj p Value** | **Sig BH Adj p** | **Effect Size** |
| --- | --- | --- | --- | --- | --- | --- | --- |
| Age range: Gender(1,777) | 0.1146 | 0.004 | 4.05 | 0.0446 | 0.0669 | . | - |
| Age range: UR Status(1,777) | 17% | 1% | 585% | 2% | 5% | * | - |
| Age range: Gender*UR Status(1,777) | 0.0972 | -0.001 | 0.28 | 0.5974 | 0.5974 |  | - |
| T1 career interest in research academic position: Gender(1,777) | 22% | 2% | 2044% | 0% | 0% | *** | (S) |
| T1 career interest in research academic position: UR Status(1,777) | -0.0712 | -0.001 | 0.09 | 0.7593 | 0.7593 |  | - |
| T1 career interest in research academic position: Gender*UR Status(1,777) | 10% | 0% | 52% | 47% | 70% |  | - |
| T1 career interest in teaching academic position: Gender(1,777) | -0.004 | -0.001 | 0.15 | 0.699 | 0.699 |  | - |
| T1 career interest in teaching academic position: UR Status(1,777) | -0.0453 | -0.001 | 0.22 | 0.6381 | 0.699 |  | - |
| T1 career interest in teaching academic position: Gender*UR Status(1,777) | 0.1793 | 0 | 0.99 | 0.3197 | 0.699 |  | - |
| T1 career interest in non-academic research: Gender(1,777) | -0.0205 | -0.001 | 0.07 | 0.7885 | 0.9452 |  | - |
| T1 career interest in non-academic research: UR Status(1,777) | 0.0017 | -0.001 | 0.01 | 0.9319 | 0.9452 |  | - |
| T1 career interest in non-academic research: Gender*UR Status(1,777) | 0.0128 | -0.001 | 0 | 0.9452 | 0.9452 |  | - |
| T1 career interest in science-related non-research: Gender(1,777) | -0.2029 | 0.018 | 15.55 | 0.0001 | 0.0003 | *** | (S) |
| T1 career interest in science-related non-research: UR Status(1,777) | 0.2492 | 0.002 | 2.29 | 0.1303 | 0.1312 |  | - |
| T1 career interest in science-related non-research: Gender*UR Status(1,777) | -0.2517 | 0.002 | 2.28 | 0.1312 | 0.1312 |  | - |
| T1 career interest in other non-science-related: Gender(1,777) | 0.0926 | 0.001 | 1.42 | 0.2342 | 0.3513 |  | - |
| T1 career interest in other non-science-related: UR Status(1,777) | 0.1195 | -0.001 | 0.05 | 0.8276 | 0.8276 |  | - |
| T1 career interest in other non-science-related: Gender*UR Status(1,777) | -0.2142 | 0.002 | 2.5 | 0.1145 | 0.3435 |  | - |
| (Fac) PhD belongingness department/social: Gender(1,777) | -0.0489 | -0.001 | 0.13 | 0.7148 | 0.7148 |  | - |
| (Fac) PhD belongingness department/social: UR Status(1,777) | -0.4044 | 0.002 | 2.74 | 0.0984 | 0.1476 |  | - |
| (Fac) PhD belongingness department/social: Gender*UR Status(1,777) | 0.4962 | 0.007 | 6.45 | 0.0113 | 0.0339 | * | - |
| Support from faculty at PhD institution: Gender(1,777) | 0.0536 | 0.002 | 2.8 | 0.0947 | 0.1651 |  | - |
| Support from faculty at PhD institution: UR Status(1,777) | -0.2094 | 0.001 | 1.93 | 0.1651 | 0.1651 |  | - |
| Support from faculty at PhD institution: Gender*UR Status(1,777) | 0.2256 | 0.002 | 2.5 | 0.1142 | 0.1651 |  | - |
| Support from faculty outside PhD institution: Gender(1,777) | 0.0932 | 0.001 | 2.12 | 0.1461 | 0.21915 |  | - |
| Support from faculty outside PhD institution: UR Status(1,777) | 0.2455 | 0.008 | 7.23 | 0.0073 | 0.0219 | * | - |
| Support from faculty outside PhD institution: Gender*UR Status(1,777) | 0.0784 | -0.001 | 0.14 | 0.7105 | 0.7105 |  | - |
| Career advice: PhD Advisor: Gender(1,777) | 0.104 | 0.007 | 6.85 | 0.009 | 0.027 | * | - |
| Career advice: PhD Advisor: UR Status(1,777) | -0.2578 | 0.001 | 1.91 | 0.1677 | 0.1677 |  | - |
| Career advice: PhD Advisor: Gender*UR Status(1,777) | 0.3056 | 0.004 | 3.79 | 0.0518 | 0.0777 | . | - |
| Career advice: PhD Department/Program: Gender(1,777) | 0.0453 | 0.001 | 1.69 | 0.1943 | 0.2393 |  | - |
| Career advice: PhD Department/Program: UR Status(1,777) | 0.0242 | 0.001 | 2.08 | 0.1498 | 0.2393 |  | - |
| Career advice: PhD Department/Program: Gender*UR Status(1,777) | 0.1969 | 0 | 1.39 | 0.2393 | 0.2393 |  | - |
| # of skills agreed program taught: Gender(1,777) | -0.1161 | -0.001 | 0.48 | 0.4897 | 0.73455 |  | - |
| # of skills agreed program taught: UR Status(1,777) | -0.4903 | 0.003 | 3.54 | 0.0604 | 0.1812 |  | - |
| # of skills agreed program taught: Gender*UR Status(1,777) | -0.11 | -0.001 | 0.04 | 0.8493 | 0.8493 |  | - |
| T1->T2 interest change in research academia: Gender(1,777) | 0.123 | 0.004 | 3.75 | 0.0533 | 0.0876 | . | - |
| T1->T2 interest change in research academia: UR Status(1,777) | -0.204 | 0.003 | 3.59 | 0.0584 | 0.0876 | . | - |
| T1->T2 interest change in research academia: Gender*UR Status(1,777) | 0.0634 | -0.001 | 0.12 | 0.7285 | 0.7285 |  | - |
| T1->T2 interest change in teaching academia: Gender(1,777) | -0.0778 | -0.001 | 0.57 | 0.4501 | 0.67515 |  | - |
| T1->T2 interest change in teaching academia: UR Status(1,777) | -0.0936 | -0.001 | 0.01 | 0.938 | 0.938 |  | - |
| T1->T2 interest change in teaching academia: Gender*UR Status(1,777) | 0.2045 | 0.001 | 1.56 | 0.2127 | 0.6381 |  | - |
| T1->T2 interest change in non-academic research: Gender(1,777) | 0.0978 | 0.003 | 3.54 | 0.0604 | 0.1722 |  | - |
| T1->T2 interest change in non-academic research: UR Status(1,777) | 0.0946 | 0.002 | 2.49 | 0.1148 | 0.1722 |  | - |
| T1->T2 interest change in non-academic research: Gender*UR Status(1,777) | 0.0688 | -0.001 | 0.18 | 0.6722 | 0.6722 |  | - |
| T1->T2 interest change in science non-research: Gender(1,777) | -0.3138 | 0.021 | 17.91 | 0 | 0 | *** | (S) |
| T1->T2 interest change in science non-research: UR Status(1,777) | 0.0728 | 0.01 | 9.13 | 0.0026 | 0.0039 | ** | (S) |
| T1->T2 interest change in science non-research: Gender*UR Status(1,777) | 0.3545 | 0.005 | 4.73 | 0.0299 | 0.0299 | * | - |
| T1->T2 interest change in non-science/other: Gender(1,777) | -0.0054 | -0.001 | 0.05 | 0.829 | 0.829 |  | - |
| T1->T2 interest change in non-science/other: UR Status(1,777) | 0.1709 | 0.004 | 4.38 | 0.0367 | 0.1101 |  | - |
| T1->T2 interest change in non-science/other: Gender*UR Status(1,777) | -0.0555 | -0.001 | 0.16 | 0.6866 | 0.829 |  | - |
| (Fac) Postdoc advisor relationship: Gender(1,574) | 0.1543 | 0.008 | 5.56 | 0.0187 | 0.0561 | . | - |
| (Fac) Postdoc advisor relationship: UR Status(1,574) | -0.1977 | -0.001 | 0.47 | 0.4913 | 0.4913 |  | - |
| (Fac) Postdoc advisor relationship: Gender*UR Status(1,574) | 0.2354 | 0 | 1.17 | 0.2795 | 0.41925 |  | - |
| (Fac) Postdoc belongingness department/social: Gender(1,574) | 0.1811 | 0.01 | 6.73 | 0.0097 | 0.0291 | * | (S) |
| (Fac) Postdoc belongingness department/social: UR Status(1,574) | -0.2456 | 0 | 1.06 | 0.3027 | 0.3027 |  | - |
| (Fac) Postdoc belongingness department/social: Gender*UR Status(1,574) | 0.2477 | 0 | 1.2 | 0.2728 | 0.3027 |  | - |
| Support from faculty at Postdoc institution: Gender(1,574) | 0.1631 | 0.006 | 4.72 | 0.0302 | 0.0906 | . | - |
| Support from faculty at Postdoc institution: UR Status(1,574) | -0.0093 | -0.002 | 0.01 | 0.9392 | 0.9392 |  | - |
| Support from faculty at Postdoc institution: Gender*UR Status(1,574) | 0.0332 | -0.002 | 0.02 | 0.8753 | 0.9392 |  | - |
| Support from faculty outside Postdoc institution: Gender(1,574) | 0.1701 | 0.003 | 2.73 | 0.0988 | 0.1482 |  | - |
| Support from faculty outside Postdoc institution: UR Status(1,574) | 0.3155 | 0.003 | 2.88 | 0.0902 | 0.1482 |  | - |
| Support from faculty outside Postdoc institution: Gender*UR Status(1,574) | -0.2138 | 0 | 0.79 | 0.3744 | 0.3744 |  | - |
| Career advice: Postdoc Advisor: Gender(1,550) | -0.1115 | -0.001 | 0.33 | 0.565 | 0.565 |  | - |
| Career advice: Postdoc Advisor: UR Status(1,550) | -0.1419 | -0.001 | 0.57 | 0.4519 | 0.565 |  | - |
| Career advice: Postdoc Advisor: Gender*UR Status(1,550) | 0.4138 | 0.005 | 3.98 | 0.0466 | 0.1398 |  | - |
| Career advice: Postdoc Department/Program: Gender(1,461) | -0.0397 | -0.002 | 0 | 0.9487 | 0.9487 |  | - |
| Career advice: Postdoc Department/Program: UR Status(1,461) | 0.0285 | 0.003 | 2.29 | 0.1305 | 0.3915 |  | - |
| Career advice: Postdoc Department/Program: Gender*UR Status(1,461) | 0.2215 | 0.001 | 1.26 | 0.2613 | 0.39195 |  | - |
| Career advice: Postdoc Institution: Gender(1,491) | -0.0806 | 0 | 0.75 | 0.3855 | 0.57825 |  | - |
| Career advice: Postdoc Institution: UR Status(1,491) | 0.1518 | 0.004 | 3.06 | 0.081 | 0.243 |  | - |
| Career advice: Postdoc Institution: Gender*UR Status(1,491) | 0.0381 | -0.002 | 0.04 | 0.8468 | 0.8468 |  | - |
| (Fac) Postdoc skills training: Supervision/Leadership: Gender(1,574) | 0.155 | 0.006 | 4.53 | 0.0337 | 0.1011 |  | - |
| (Fac) Postdoc skills training: Supervision/Leadership: UR Status(1,574) | -0.2116 | 0.002 | 2.04 | 0.1538 | 0.2307 |  | - |
| (Fac) Postdoc skills training: Supervision/Leadership: Gender*UR Status(1,574) | 0.1157 | -0.001 | 0.3 | 0.5853 | 0.5853 |  | - |
| Years of research prior to PhD program?: Gender(1,777) | -0.0721 | -0.001 | 0.08 | 0.7836 | 0.7836 |  | - |
| Years of research prior to PhD program?: UR Status(1,777) | 0.1531 | 0.001 | 1.71 | 0.1918 | 0.5754 |  | - |
| Years of research prior to PhD program?: Gender*UR Status(1,777) | 0.184 | -0.001 | 0.24 | 0.6213 | 0.7836 |  | - |
| Years in formal post-baccalaureate research program: Gender(1,777) | 0.0807 | 0.002 | 2.92 | 0.0881 | 0.13215 |  | - |
| Years in formal post-baccalaureate research program: UR Status(1,777) | -0.0815 | 0 | 1.03 | 0.3114 | 0.3114 |  | - |
| Years in formal post-baccalaureate research program: Gender*UR Status(1,777) | 0.4143 | 0.003 | 3 | 0.0838 | 0.13215 |  | - |
| Years it took to complete PhD?: Gender(1,777) | -0.1554 | 0.005 | 5.15 | 0.0235 | 0.03525 | * | - |
| Years it took to complete PhD?: UR Status(1,777) | 0.4405 | 0.013 | 11.4 | 0.0008 | 0.0024 | ** | (S) |
| Years it took to complete PhD?: Gender*UR Status(1,777) | -0.1733 | 0 | 0.68 | 0.4115 | 0.4115 |  | - |
| Years since completed PhD: Gender(1,777) | 0.4702 | 0.011 | 9.85 | 0.0018 | 0.0054 | ** | (S) |
| Years since completed PhD: UR Status(1,777) | -0.2277 | -0.001 | 0.09 | 0.7641 | 0.7641 |  | - |
| Years since completed PhD: Gender*UR Status(1,777) | 0.3262 | -0.001 | 0.52 | 0.4726 | 0.7089 |  | - |
| # of postdoc positions?: Gender(1,777) | -0.0964 | 0.001 | 1.64 | 0.2003 | 0.2003 |  | - |
| # of postdoc positions?: UR Status(1,777) | -0.0045 | 0.001 | 1.78 | 0.1823 | 0.2003 |  | - |
| # of postdoc positions?: Gender*UR Status(1,777) | 0.1942 | 0.001 | 2.05 | 0.1526 | 0.2003 |  | - |
| First-author peer-reviewed publications you have?: Gender(1,777) | 0.0778 | 0.012 | 10.26 | 0.0014 | 0.0021 | ** | (S) |
| First-author peer-reviewed publications you have?: UR Status(1,777) | -0.1069 | 0.012 | 10.83 | 0.001 | 0.0021 | ** | (S) |
| First-author peer-reviewed publications you have?: Gender*UR Status(1,777) | 0.0013 | -0.001 | 0 | 0.9838 | 0.9838 |  | - |
| (Fac) Like structural aspects of academia: Gender(1,777) | 0.2821 | 0.023 | 19.26 | 0 | 0 | *** | (S) |
| (Fac) Like structural aspects of academia: UR Status(1,777) | 0.0165 | -0.001 | 0.01 | 0.9222 | 0.9261 |  | - |
| (Fac) Like structural aspects of academia: Gender*UR Status(1,777) | -0.0162 | -0.001 | 0.01 | 0.9261 | 0.9261 |  | - |
| (Fac) Like academic teaching/mentoring: Gender(1,777) | -0.0487 | -0.001 | 0.31 | 0.5791 | 0.5851 |  | - |
| (Fac) Like academic teaching/mentoring: UR Status(1,777) | 0.0611 | 0.001 | 1.69 | 0.1946 | 0.5838 |  | - |
| (Fac) Like academic teaching/mentoring: Gender*UR Status(1,777) | 0.0874 | -0.001 | 0.3 | 0.5851 | 0.5851 |  | - |
| Confident being independent researcher: Gender(1,777) | 0.4262 | 0.044 | 37.14 | 0 | 0 | *** | (S) |
| Confident being independent researcher: UR Status(1,777) | -0.0163 | 0 | 0.82 | 0.365 | 0.365 |  | - |
| Confident being independent researcher: Gender*UR Status(1,777) | 0.2266 | 0 | 1.18 | 0.2777 | 0.365 |  | - |
| Times supported by NIH, pre-Curr Pos: Gender(1,777) | -0.0363 | -0.001 | 0.17 | 0.6771 | 0.6771 |  | - |
| Times supported by NIH, pre-Curr Pos: UR Status(1,777) | -0.3254 | 0.011 | 9.54 | 0.0021 | 0.0063 | ** | (S) |
| Times supported by NIH, pre-Curr Pos: Gender*UR Status(1,777) | 0.0966 | -0.001 | 0.29 | 0.5918 | 0.6771 |  | - |
