# Supplementary material for: A Retrospective Analysis of Career Outcomes in Neuroscience
Source: eNeuro. 2024 May 24;11(5):ENEURO.0054-24.2024. doi: 10.1523/ENEURO.0054-24.2024 (PMC11134307; doi:10.1523/ENEURO.0054-24.2024)
Supplement: Figure 2-2 — Logistic Regression Results for Binomial Categorical Dependent Variables by Gender and UR Status. Results from eighteen two-way (Gender by UR Status) logistic regressions on dichotomous explanatory variables (dependent variable) to ascertain whether there were Gender or UR Status differences in the explanatory variables. UR=Under-Represented, BH Adj=Benjamini and Hochberg adjusted, Sig=Significance. Effect size: (-) = negligible effect size, (S) = small effect size, (M) = medium effect size. * = p < 0.05, *** = p < 0.001. Download Figure 2-2, DOCX file. [file eneuro-11-ENEURO.0054-24.2024-s003.docx]

Figure 2-2: Logistic Regression Results for Binomial Categorical Dependent Variables by Gender and UR Status. Results from eighteen two-way (Gender by UR Status) logistic regressions on dichotomous explanatory variables (dependent variable) to ascertain whether there were Gender or UR Status differences in the explanatory variables. UR=Under-Represented, BH Adj=Benjamini and Hochberg adjusted, Sig=Significance. Effect size: (-) = negligible effect size, (S) = small effect size, (M) = medium effect size. * = p < 0.05, *** = p < 0.001.

| **(Logistic Regression Terms groupd by analysis) Dependent Variable: Independent Variable(s)** | **Odds Ratio** | **z score** | **Raw p Value** | **BH Adj p Value** | **Sig BH Adj p** | **Effect Size** |
| --- | --- | --- | --- | --- | --- | --- |
| Do you have a disability?: Gender | 1.933 | 1.34 | 0.1786 | 0.2416 |  | (S) |
| Do you have a disability?: UR Status | 2.564 | 1.34 | 0.1812 | 0.2416 |  | (M) |
| Do you have a disability?: Gender*UR Status | 0.345 | -1.01 | 0.3118 | 0.3118 |  | (M) |
| First person/generation to graduate from 4yr college?: Gender | 1.149 | 0.71 | 0.4805 | 0.4805 |  | - |
| First person/generation to graduate from 4yr college?: UR Status | 3.601 | 4.44 | 0 | 0 | *** | (M) |
| First person/generation to graduate from 4yr college?: Gender*UR Status | 0.596 | -1.24 | 0.2163 | 0.2884 |  | (S) |
| Been a postdoctoral fellow?: Gender | 0.837 | -1 | 0.3151 | 0.3248 |  | - |
| Been a postdoctoral fellow?: UR Status | 0.743 | -0.98 | 0.3248 | 0.3248 |  | - |
| Been a postdoctoral fellow?: Gender*UR Status | 2.401 | 1.87 | 0.0609 | 0.1218 |  | (S) |
| Master's in biomed research discipline before PhD program?: Gender | 1.159 | 0.66 | 0.5075 | 0.6138 |  | - |
| Master's in biomed research discipline before PhD program?: UR Status | 1.523 | 1.18 | 0.2375 | 0.475 |  | (S) |
| Master's in biomed research discipline before PhD program?: Gender*UR Status | 1.279 | 0.5 | 0.6138 | 0.6138 |  | - |
| Undergraduate Institution= Top 50: Gender | 0.941 | -0.29 | 0.7738 | 0.8556 |  | - |
| Undergraduate Institution= Top 50: UR Status | 0.525 | -1.43 | 0.1541 | 0.3082 |  | (S) |
| Undergraduate Institution= Top 50: Gender*UR Status | 0.885 | -0.18 | 0.8556 | 0.8556 |  | - |
| Doctoral Institution= Top 50: Gender | 1.311 | 1.73 | 0.0845 | 0.338 |  | - |
| Doctoral Institution= Top 50: UR Status | 0.763 | -0.97 | 0.3319 | 0.6638 |  | - |
| Doctoral Institution= Top 50: Gender*UR Status | 1.165 | 0.39 | 0.6997 | 0.6997 |  | - |
| Important aspects of careers: Low stress: Gender | 0.722 | -0.93 | 0.3504 | 0.3628 |  | - |
| Important aspects of careers: Low stress: UR Status | 1.57 | 0.94 | 0.348 | 0.3628 |  | (S) |
| Important aspects of careers: Low stress: Gender*UR Status | 0.438 | -0.91 | 0.3628 | 0.3628 |  | (S) |
| Important aspects of careers: High autonomy: Gender | 2.252 | 5.07 | 0 | 0 | *** | (S) |
| Important aspects of careers: High autonomy: UR Status | 0.571 | -1.77 | 0.0769 | 0.102533 |  | (S) |
| Important aspects of careers: High autonomy: Gender*UR Status | 0.785 | -0.56 | 0.5731 | 0.5731 |  | - |
| Important aspects of careers: Work-life balance: Gender | 0.541 | -3.87 | 0.0001 | 0.0004 | *** | (S) |
| Important aspects of careers: Work-life balance: UR Status | 1.344 | 1.03 | 0.3015 | 0.3015 |  | - |
| Important aspects of careers: Work-life balance: Gender*UR Status | 0.615 | -1.2 | 0.2316 | 0.3015 |  | (S) |
| Important aspects of careers: Personal competence: Gender | 1.014 | 0.08 | 0.9324 | 0.9324 |  | - |
| Important aspects of careers: Personal competence: UR Status | 0.816 | -0.71 | 0.4764 | 0.9324 |  | - |
| Important aspects of careers: Personal competence: Gender*UR Status | 1.163 | 0.37 | 0.709 | 0.9324 |  | - |
| Important aspects of careers: Collaboration: Gender | 1.085 | 0.51 | 0.612 | 0.612 |  | - |
| Important aspects of careers: Collaboration: UR Status | 0.653 | -1.4 | 0.1616 | 0.3232 |  | (S) |
| Important aspects of careers: Collaboration: Gender*UR Status | 1.376 | 0.76 | 0.45 | 0.6 |  | - |
| Important aspects of careers: Job market: Gender | 0.884 | -0.63 | 0.5284 | 0.7942 |  | - |
| Important aspects of careers: Job market: UR Status | 1.09 | 0.26 | 0.7942 | 0.7942 |  | - |
| Important aspects of careers: Job market: Gender*UR Status | 1.265 | 0.5 | 0.6155 | 0.7942 |  | - |
| Important aspects of careers: Geographic location: Gender | 0.64 | -2.63 | 0.0085 | 0.017 | * | (S) |
| Important aspects of careers: Geographic location: UR Status | 0.745 | -0.99 | 0.3209 | 0.3209 |  | - |
| Important aspects of careers: Geographic location: Gender*UR Status | 2.293 | 1.99 | 0.0462 | 0.0616 | . | (S) |
| Important aspects of careers: Job security: Gender | 0.96 | -0.23 | 0.8163 | 0.8163 |  | - |
| Important aspects of careers: Job security: UR Status | 1.827 | 2.13 | 0.0331 | 0.0662 | . | (S) |
| Important aspects of careers: Job security: Gender*UR Status | 0.43 | -1.94 | 0.0522 | 0.0696 | . | (S) |
| Important aspects of careers: Monetary compensation: Gender | 1.281 | 1.5 | 0.1333 | 0.177733 |  | - |
| Important aspects of careers: Monetary compensation: UR Status | 2.158 | 2.77 | 0.0057 | 0.0114 | * | (S) |
| Important aspects of careers: Monetary compensation: Gender*UR Status | 0.807 | -0.54 | 0.5882 | 0.5882 |  | - |
| Important aspects of careers: Make a difference: Gender | 0.927 | -0.48 | 0.6337 | 0.6337 |  | - |
| Important aspects of careers: Make a difference: UR Status | 0.794 | -0.83 | 0.4046 | 0.539467 |  | - |
| Important aspects of careers: Make a difference: Gender*UR Status | 1.461 | 0.95 | 0.341 | 0.539467 |  | - |
| Important aspects of careers: Varied, diverse work: Gender | 0.737 | -1.78 | 0.0755 | 0.151 |  | - |
| Important aspects of careers: Varied, diverse work: UR Status | 0.873 | -0.46 | 0.647 | 0.647 |  | - |
| Important aspects of careers: Varied, diverse work: Gender*UR Status | 1.285 | 0.58 | 0.559 | 0.647 |  | - |
| Important aspects of careers: Intellectually stimulating: Gender | 1.44 | 1.88 | 0.0606 | 0.1212 |  | - |
| Important aspects of careers: Intellectually stimulating: UR Status | 0.622 | -1.61 | 0.108 | 0.1416 |  | (S) |
| Important aspects of careers: Intellectually stimulating: Gender*UR Status | 2.055 | 1.47 | 0.1416 | 0.1416 |  | (S) |
