# Supplementary material for: A Retrospective Analysis of Career Outcomes in Neuroscience
Source: eNeuro. 2024 May 24;11(5):ENEURO.0054-24.2024. doi: 10.1523/ENEURO.0054-24.2024 (PMC11134307; doi:10.1523/ENEURO.0054-24.2024)
Supplement: Figure 2-3 — Multinomial Logistic Regression Results for Multinomial Categorical Dependent Variables by Gender and UR Status. Results from three two-way (Gender by UR Status) multinomial logistic regressions on multinomial categorical explanatory variables (dependent variable) to ascertain whether there were Gender or UR Status differences in the explanatory variables. UR=Under-Represented, Sig=Significance. * = p < 0.05, *** = p < 0.001. Download Figure 2-3, DOCX file. [file eneuro-11-ENEURO.0054-24.2024-s004.docx]

Figure 2-3: Multinomial Logistic Regression Results for Multinomial Categorical Dependent Variables by Gender and UR Status. Results from three two-way (Gender by UR Status) multinomial logistic regressions on multinomial categorical explanatory variables (dependent variable) to ascertain whether there were Gender or UR Status differences in the explanatory variables. UR=Under-Represented, Sig=Significance. * = p < 0.05, *** = p < 0.001.

| **(Multinomial Logistic Regression Terms groupd by analysis) Dependent Variable: Independent Variable(s)** | **Liklihood Ratio** | **df** | **p Value** | **Sig** |
| --- | --- | --- | --- | --- |
| Career goal changed research to non-research?: Gender | 0.0045 | 0 | 0 | *** |
| Career goal changed research to non-research?: UR Status | -0.0043 | 0 | 1 |  |
| Career goal changed research to non-research?: Gender*UR Status | 4.7617 | 2 | 0.0925 | . |
| How found current position?: Gender | 0 | 0 | 1 |  |
| How found current position?: UR Status | 0 | 0 | 1 |  |
| How found current position?: Gender*UR Status | 11.1963 | 5 | 0.0476 | * |
| In what discipline is/will be PhD?: Gender | 0 | 0 | 0 | *** |
| In what discipline is/will be PhD?: UR Status | 0 | 0 | 0 | *** |
| In what discipline is/will be PhD?: Gender*UR Status | 0.2171 | 3 | 0.9748 |  |
