# Supplementary material for: A Retrospective Analysis of Career Outcomes in Neuroscience
Source: eNeuro. 2024 May 24;11(5):ENEURO.0054-24.2024. doi: 10.1523/ENEURO.0054-24.2024 (PMC11134307; doi:10.1523/ENEURO.0054-24.2024)
Supplement: Figure 4-1 — Lasso regression predicting Academia vs. Not Academia. Results of 5-fold cross-validated lasso logistic regression predicting whether respondents were currently in academic or non-academic positions from all explanatory variables and their interactions with Gender and/or UR Status. Conf=confidence, CV=cross-validation, sd=standard deviation. Download Figure 4-1, DOCX file. [file eneuro-11-ENEURO.0054-24.2024-s005.docx]

Figure 4-1: Lasso regression predicting Academia vs. Not Academia. Results of 5-fold cross-validated lasso logistic regression predicting whether respondents were currently in academic or non-academic positions from all explanatory variables and their interactions with Gender and/or UR Status. Conf=confidence, CV=cross-validation, sd=standard deviation.

| **Dependent Variable** | (dichotomous) Current position in academia or not | | |  |  |  |
| --- | --- | --- | --- | --- | --- | --- |
| **Independent Variables** | All explanatory variables |  |  |  |  |  |
|  | All interactions of explanatory variables with Gender, UR Status, and their interaction | | | | | |

| Call: glinternet.cv(X = x_mat, Y = why$Q1_Academia_n, numLevels = nlvl, |
| --- |
| nFolds = 5, nLambda = 100, lambdaMinRatio = 0.001, interactionCandidates = c(1:3), |
| family = "binomial") |
| Results of 5 -fold cross validation:  Minimum CV error of 0.3593397 at lambda = 0.0003121563  Chosen lambda (l1sd+3) = 0.000584923996687757 |

| **Remaining Categorical Main Effects** | **Coefficients** | |
| --- | --- | --- |
| Important aspects of careers: High autonomy | (not selected) | (selected) |
|  | -0.17 | 0.17 |
| Important aspects of careers: Work-life balance | (not selected) | (selected) |
|  | 0.02 | -0.02 |
| Important aspects of careers: Job security | (not selected) | (selected) |
|  | -0.07 | 0.07 |
| Important aspects of careers: Monetary compensation | (not selected) | (selected) |
|  | 0.63 | -0.63 |
| Important aspects of careers: Varied, diverse work | (not selected) | (selected) |
|  | 0.22 | -0.22 |
| Gender | Female | Male |
|  | -0.10 | 0.05 |

| **Remaining Continuous Main Effects** | **Coefficients** |
| --- | --- |
| T1 career interest in research academic | 0.19 |
| T1 career interest in non-academic research | -0.31 |
| T1 career interest in science-related non-research | -0.06 |
| (Fac) PhD belongingness department/social | -0.05 |
| T1->T2 interest change in research academia | 0.33 |
| T1->T2 interest change in teaching academia | 0.28 |
| T1->T2 interest change in non-academic research | -0.18 |
| T1->T2 interest change in science non-research | -0.08 |
| (Fac) Postdoc advisor relationship | 0.03 |
| Postdoc Support, Faculty at primary institution | 0.23 |
| Postdoc Career advice, Advisor | -0.07 |
| Postdoc Career advice, Institution | -0.24 |
| Years it took to complete PhD? | 0.00 |
| Years since completed PhD | 0.05 |
| First-author publication rate | 0.44 |
| (Fac) Like structural aspects of academia | 0.78 |
| (Fac) Like academic teaching/mentoring | 0.14 |
| Career goal changed? No, still is research-based | 0.67 |
| Career goal changed? Yes, changed away from research | -0.13 |
| (Fac) Postdoc belongingness department/social | 0.00 |

| <NONE> |  |  |  |  |
| --- | --- | --- | --- | --- |
|  |  |  |  |  |
| **Remaining Continuous/Continuous Interactions** |  |  |  |  |
| <NONE> |  |  |  |  |
|  |  |  |  |  |
| **Remaining Categorical/Continuous Interactions** |  |  |  |  |
| Gender*(Fac) Postdoc belongingness department/social |  |  |  |  |
| Gender*Postdoc Support, Faculty at primary institution |  |  |  |  |
|  |  |  |  |  |
| **Overall Equation Measures** | **estimate** | **conf low** | **conf high** | **p value** |
| accuracy | 87% | 85% | 90% | 0.0000 |
| kappa | 0.72 |  | | |
| mcnemar | 0.0339 | | | |
| sensitivity | 0.85 |  |  |  |
| specificity | 0.88 |  |  |  |
| pos_pred_value | 0.79 |  |  |  |
| neg_pred_value | 0.92 |  |  |  |
| precision | 0.79 |  |  |  |
| recall | 0.85 |  |  |  |
| f1 | 0.82 |  |  |  |
| prevalence | 0.34 |  |  |  |
| detection_rate | 0.29 |  |  |  |
| detection_prevalence | 0.36 |  |  |  |
| balanced_accuracy | 0.87 |  |  |  |
