# Supplementary material for: A Retrospective Analysis of Career Outcomes in Neuroscience
Source: eNeuro. 2024 May 24;11(5):ENEURO.0054-24.2024. doi: 10.1523/ENEURO.0054-24.2024 (PMC11134307; doi:10.1523/ENEURO.0054-24.2024)
Supplement: Figure 5-1 — Lasso regression predicting Research Academia vs. Teaching Academia. Results of 5-fold cross-validated lasso logistic regression predicting whether respondents were currently in research or teaching academic positions from all explanatory variables and their interactions with Gender and/or UR Status. Conf=confidence, CV=cross-validation, sd=standard deviation. Download Figure 5-1, DOCX file. [file eneuro-11-ENEURO.0054-24.2024-s006.docx]

Figure 5-1: Lasso regression predicting Research Academia vs. Teaching Academia. Results of 5-fold cross-validated lasso logistic regression predicting whether respondents were currently in research or teaching academic positions from all explanatory variables and their interactions with Gender and/or UR Status. Conf=confidence, CV=cross-validation, sd=standard deviation.

| **Dependent Variable** | (dichotomous) Current position Research Academia vs. Teaching Academia |
| --- | --- |
| **Independent Variables** | All explanatory variables |
|  | All interactions of explanatory variables with Gender, UR Status, and their interaction |

| Call: glinternet.cv(X = x_mat, Y = Q2, numLevels = nlvl, nFolds = 5, |
| --- |
| nLambda = 100, lambdaMinRatio = 0.001, interactionCandidates = c(1:3), |
| family = "binomial") |
| Results of 5 -fold cross validation:  Minimum CV error of 0.2988984 at lambda = 0.0007375471  Chosen lambda (l1sd+10) = 0.00112100605366625 |

| **Remaining Categorical Main Effects** | **Coefficients** | |
| --- | --- | --- |
| Important aspects of careers: Collaboration | (not selected) | (selected) |
|  | -0.10 | 0.10 |
| Important aspects of careers: Job security | (not selected) | (selected) |
|  | 0.04 | -0.04 |
| Gender | Female | Male |
|  | -0.04 | 0.04 |
| Important aspects of careers: Intellectually stimulating | (not selected) | (selected) |
|  | -0.03 | 0.03 |

| **Remaining Continuous Main Effects** | **Coefficients** |
| --- | --- |
| T1 career interest in research academic | 0.60 |
| T1 career interest in teaching academic | -0.53 |
| T1->T2 interest change in research academia | 0.27 |
| T1->T2 interest change in teaching academia | -0.63 |
| (Fac) Like academic teaching/mentoring | -0.77 |
| Career goal changed? No, still is research-based | 1.62 |
| Times supported by NIH, pre-Curr Pos | -0.01 |

| **Remaining Categorical/Categorical Interactions** |  |  |  |  |
| --- | --- | --- | --- | --- |
| Gender*Important aspects of careers: Intellectually stimulating |  |  |  |  |
|  |  |  |  |  |
| **Remaining Continuous/Continuous Interactions** |  |  |  |  |
| <NONE> |  |  |  |  |
|  |  |  |  |  |
| **Remaining Categorical/Continuous Interactions** |  |  |  |  |
| <NONE> |  |  |  |  |
| **Overall Equation Measures** | **estimate** | **conf low** | **conf high** | **p value** |
| accuracy | 88% | 85% | 91% | 0.3737 |
| kappa | 0.57 |  | | |
| mcnemar | 0.0000 | | | |
| sensitivity | 0.83 |  |  |  |
| specificity | 0.89 |  |  |  |
| pos_pred_value | 0.51 |  |  |  |
| neg_pred_value | 0.97 |  |  |  |
| precision | 0.51 |  |  |  |
| recall | 0.83 |  |  |  |
| f1 | 0.63 |  |  |  |
| prevalence | 0.13 |  |  |  |
| detection_rate | 0.10 |  |  |  |
| detection_prevalence | 0.20 |  |  |  |
| balanced_accuracy | 0.86 |  |  |  |
