# Supplementary material for: A Retrospective Analysis of Career Outcomes in Neuroscience
Source: eNeuro. 2024 May 24;11(5):ENEURO.0054-24.2024. doi: 10.1523/ENEURO.0054-24.2024 (PMC11134307; doi:10.1523/ENEURO.0054-24.2024)
Supplement: Figure 6-1 — Lasso regression predicting Non-academic Research vs. Science/Non-research. Results of 5-fold cross-validated lasso logistic regression predicting whether respondents were currently in non-academic research or scitentific non-research positions from all explanatory variables and their interactions with Gender and/or UR Status. Conf=confidence, CV=cross-validation, sd=standard deviation. Download Figure 6-1, DOCX file. [file eneuro-11-ENEURO.0054-24.2024-s007.docx]

Figure 6-1: Lasso regression predicting Non-academic Research vs. Science/Non-research. Results of 5-fold cross-validated lasso logistic regression predicting whether respondents were currently in non-academic research or scitentific non-research positions from all explanatory variables and their interactions with Gender and/or UR Status. Conf=confidence, CV=cross-validation, sd=standard deviation.

| **Dependent Variable** | (dichotomous) Current position Non-academic research vs. Science/Non-research |
| --- | --- |
| **Independent Variables** | All explanatory variables |
|  | All interactions of explanatory variables with Gender, UR Status, and their interaction |

| Call: glinternet.cv(X = x_mat, Y = Q3, numLevels = nlvl, nFolds = 5, |
| --- |
| nLambda = 100, lambdaMinRatio = 0.001, interactionCandidates = c(1:3), |
| family = "binomial") |
| Results of 5 -fold cross validation:  Minimum CV error of 0.3873769 at lambda = 0.001106901  Chosen lambda (l1sd+7) = 0.00479150820375284 |

| **Remaining Categorical Main Effects** | **Coefficients** |
| --- | --- |
| <NONE> | <NONE> |

| **Remaining Continuous Main Effects** | **Coefficients** |
| --- | --- |
| T1->T2 interest change in non-academic research | 0.26 |
| Career goal changed? No, still is research-based | 2.15 |

| **Remaining Categorical/Categorical Interactions** |
| --- |
| Gender*Important aspects of careers: Intellectually stimulating |
|  |
| **Remaining Continuous/Continuous Interactions** |
| <NONE> |
|  |
| **Remaining Categorical/Continuous Interactions** |
| <NONE> |

| **Overall Equation Measures** | **estimate** | **conf low** | **conf high** | **p value** |
| --- | --- | --- | --- | --- |
| accuracy | 82% | 77% | 87% | 0.0000 |
| kappa | 0.64 |  | | |
| mcnemar | 0.0011 | | | |
| sensitivity | 0.79 |  |  |  |
| specificity | 0.88 |  |  |  |
| pos_pred_value | 0.91 |  |  |  |
| neg_pred_value | 0.72 |  |  |  |
| precision | 0.91 |  |  |  |
| recall | 0.79 |  |  |  |
| f1 | 0.85 |  |  |  |
| prevalence | 0.61 |  |  |  |
| detection_rate | 0.48 |  |  |  |
| detection_prevalence | 0.53 |  |  |  |
| balanced_accuracy | 0.83 |  |  |  |
